# The Pharmacokinetics, Dosage, Preparation Forms, and Efficacy of Orally Administered Melatonin for Non-Organic Sleep Disorders in Autism Spectrum Disorder During Childhood and Adolescence: A Systematic Review

**DOI:** 10.3390/children12050648

**Published:** 2025-05-16

**Authors:** Ekkehart Paditz, Bertold Renner, Rainer Koch, Barbara M. Schneider, Angelika A. Schlarb, Osman S. Ipsiroglu

**Affiliations:** 1 Centre for Applied Prevention/Zentrum für Angewandte Prävention, 01307 Dresden, Germany; 2 Institute of Clinical Pharmacology, Faculty of Medicine Carl Gustav Carus, Dresden University of Technology, 01069 Dresden, Germany; bertold.renner@tu-dresden.de; 3 Formerly Institute for Medical Informatics and Biometry, Faculty of Medicine Carl Gustav Carus, Dresden University of Technology, 01069 Dresden, Germany; rainer.koch.01@gmx.de; 4 Paediatric Sleep Medicine Centre in Foundation, Vancouver, BC V5Z 4H4, Canada; b.schneider@kinder-schlaf.de; 5 Faculty of Psychology and Sports Science, Bielefeld University, 33615 Bielefeld, Germany; angelika.schlarb@uni-bielefeld.de; 6 Interdisciplinary Sleep Program, Sleep/Wake-Behaviour Clinic, Departments of Pediatrics & Psychiatry, University of British Columbia, Vancouver, BC V6T 1Z4, Canada; oipsiroglu@bcchr.ca

**Keywords:** melatonin in children and adolescents, autism spectrum disorder, sleep, pharmacokinetics, RCT, non-delayed preparations

## Abstract

**Background:** To date, it remains unclear which oral doses and preparation forms of melatonin should be recommended for children and adolescents with non-organic sleep disorders and autism spectrum disorder (ASD). We reviewed the current state of knowledge on this topic based on randomised placebo-controlled trials (RCTs) and diagnosis-related blood melatonin concentrations available in this age group. **Method:** Two investigators independently searched PubMed, PsycINFO, MEDLINE, and Cochrane CENTRAL on 1 March 2025 for the keywords “melatonin”, “autism”, and “randomised” in titles and abstracts in all languages, including an evaluation of the references of the reviews, systematic reviews, and meta-analyses published up to that date, some of which were based on searches in numerous databases. Based on this, additional in-depth searches were carried out in PubMed for pharmacokinetic, physiological, and pathophysiological data on melatonin in children and adolescents, with a special focus on ASD. **Results**: To date, five RCTs on non-organic sleep disorders in children and adolescents with the sole diagnosis of ASD or with subgroup analyses in the presence of several initial diagnoses such as ADHD, epilepsy, Smith–Magenis, or Fragile X syndrome are available. In these studies, rapid-release, non-delayed preparations were administered orally. In one of these studies, the clinical efficacy of a combination preparation with a sustained-release and a non-released active substance component was tested. Pharmacokinetic data with multiple determinations of melatonin concentrations in the blood are only available for children with ASD in the form of a case series (N = 9). **Discussion**: RCTs comparing the efficacy of delayed melatonin preparations with non-delayed rapid-release oral preparations are not yet available. Physiological data and clinical effects documented in five RCTs indicate that non-delayed melatonin preparations with an initial rapid onset of action are effective for non-organic sleep disorders in children and adolescents with ASD. **Conclusions**: From a clinical, pharmacokinetic, and physiological point of view, the RCTs available to date and the data on melatonin concentrations in the blood of children with ASD, measured several times over 24 h, suggest that a low oral melatonin dose and a non-delayed preparation with rapid onset should be started in children and adolescents with non-organic sleep disorders in ASD, if sleep hygiene advice and psychotherapeutic interventions have not demonstrated sufficient effects.

## 1. Introduction

### 1.1. Sleep Disorders in Children and Adolescents with ASD

Sleep disorders in children and adolescents with autism spectrum disorder (ASD) have been detected in 19.7% (11.9 to 30.7%) of the individuals in eight population-based studies [1]. At 26.5% (15.4 to 41.6%), significantly higher prevalences were found in children than in adolescents (6.6%; 4.5–9.5%) [1]. At the same time, children and adolescents with ASD exhibit Attention Deficit Hyperactivity Disorder (ADHD) in 26.2% of cases, although the mean ADHD prevalence rates for ASD do not differ significantly between children and adolescents (26.2 vs. 35.4%) [1]. Anxiety disorders and intellectual disabilities are further significant psychiatric comorbidities in ASD, with prevalences of 11.1% and 22.9%, respectively. In the case of anxiety disorders, it is striking that these are observed less frequently in children (7.8%) than in adolescents (21.5%) [1].

Baldini et al. reported recently on the close connection between insomnia and depression in adolescents with psychiatric disorders (r = 0.94, *p* = 0.02) [2]. Both disorders are associated with an increased risk of suicide (ibid.). In children and adolescents with ASD, an almost 3-fold increase in suicidal risk was observed at first presentation in paediatric emergency departments (12.7% vs. 4.4% compared to children and adolescents without ASD) [3]. These data indicate that insomnia in children and adolescents with ASD should be diagnosed early (see comparison in [4]) and should be taken seriously. Treatment requires multimodal approaches with structured advice on sleep hygiene [5], advice on physical activation [6], psychological–psychiatric treatment using cognitive-behavioural therapy [7,8,9], and drug treatments, with a particular interest in melatonin if organic causes of the sleep disorder have been ruled out [10,11,12].

Sleep disorders in ASD are phenotypically characterised by difficulty falling asleep, restless sleep, nocturnal awakenings and fragmented sleep due to nocturnal arousals (53 vs. 40 vs. 34 vs. 32%; information from parents via standardised questionnaires) [13]. These symptoms were associated with daytime sleepiness in 15% of 60 children with ASD from China [14].

### 1.2. Objectives

The possibility that the psychiatric comorbidities described and the different types of sleep disorders are present simultaneously in at least some of the patient groups examined in the studies means that subgroup analyses are necessary. This aspect is taken into account in the present study. We were interested in the current state of knowledge on the use of melatonin in children and adolescents with sleep disorders in ASD.

### 1.3. Preliminary Considerations on the Dosage, Pharmaceutical Formulation and Pharmacokinetics of Orally Administered Melatonin Preparations

Since the discovery of melatonin in 1958 by Lerner [15], and the first descriptions of the circadian day–night rhythmicity of melatonin synthesis and secretion in humans in 1976 [16] and in almost all mammalia and vertrebralia since 1963 [17,18,19,20,21,22,23,24,25,26] and some other species studied to date [27,28,29,30,31], to the best of our knowledge, 36 randomised placebo-controlled trials (RCTs) on the efficacy of melatonin in non-organic sleep disorders in children and adolescents have been published since 1998 [6,11,32,33,34], demonstrating heterogeneous quality standards with regard to the recording of parameters associated with sleep [35]. These data show that the diurnal rhythmicity of melatonin concentrations represents evolutionary biological adaptations to the day–night and light–dark rhythms due to the Earth’s rotation, and that disorders of pineal melatonin synthesis and secretion can be influenced in an age- and diagnosis-related manner. Interestingly, the ability to synthesise and secrete melatonin rhythmically in the pineal gland in the dark of night only develops in humans postnatally during the first year of life [36,37].

General recommendations for the administration of melatonin for non-organic sleep disorders in children and adolescents—which also relate to the form of preparation, the dosage, the time of evening administration, and the resulting efficacy on defined parameters associated with restful sleep—are missing so far. In our view, diagnosis-related recommendations are required [11].

**Figure 1:**
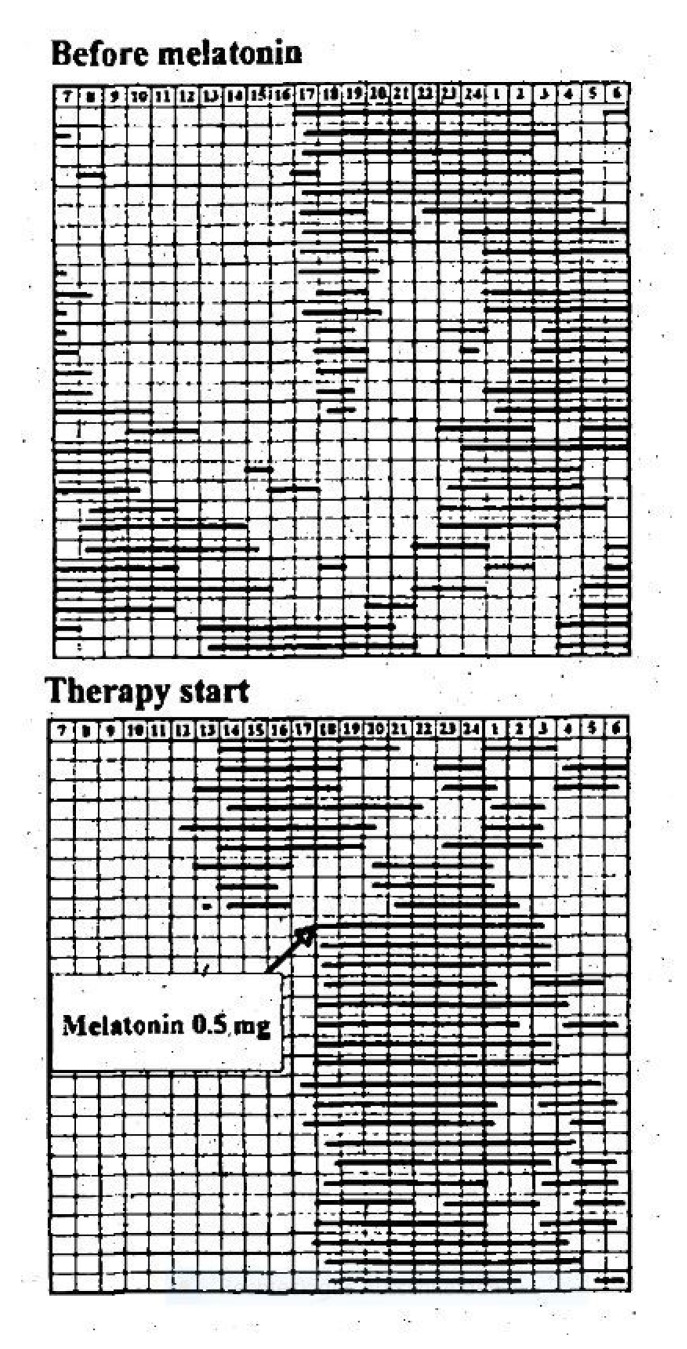
Disturbed day–night rhythm (“non-24”) in an 8-year-old blind child with microphthalmia and epilepsy after congenital toxoplasmosis reported by Palm et al. in 1991 and 1997 [39,40]. This is a graphical representation of records from the sleep diary which the parents had recorded. The black bars correspond to the visually recorded total sleep time (TST). There was normalisation of the circadian sleep–wake rhythm after the start of the treatment, comprising an evening oral administration of 0.5 mg of a rapid-release, non-delayed melatonin preparation. This study is also remarkable from a methodological point of view, as informative results were obtained on the basis of sleep logs filled out by the parents without technical appliances, which are relatively similar to today’s standard actigraphic measurements. From Palm et al., 1997 [40]. Reproduced with permission.

**Figure 2:**
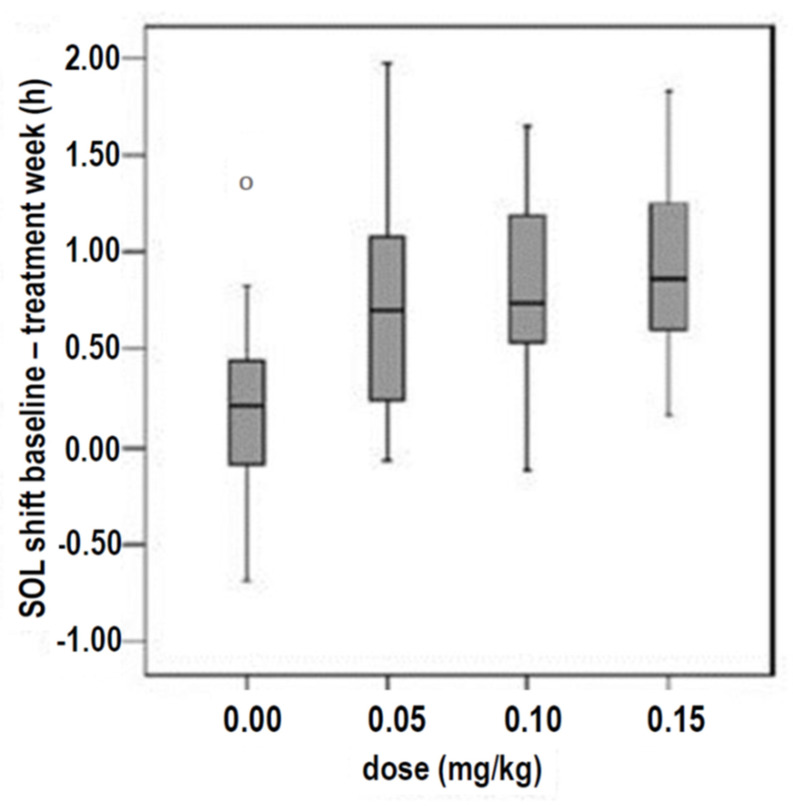
Improvement in sleep onset latency (SOL shift) after the administration of 0.05, 0.1, or 0.15 mg of melatonin per kilogram of body weight in 72 children and adolescents with delayed sleep phase disorder (DSPD) who were studied in parallel in a randomised setting compared to a placebo group (0 mg melatonin) (Van Geijlswijk et al., 2010) [42]. In this dosage range, there was no dose–response correlation in the sense of an increase in efficacy with an increase in dose (N = 16–19 per group). Reproduced with permission.

These questions of practical relevance are influenced in particular by age, the age-related elimination half-life, and underlying disorders, as follows:

- In 13 children with Angelman syndrome aged 2–10 years, **low evening oral doses of 0.3 mg** were associated with relatively high blood melatonin concentrations and improvements in actigraphic parameters, such as total sleep time (TST) [38].
- Niederhofer et al. showed in 2003 that **0.3 mg of melatonin in the non-delayed form** led to an improvement in sleep parameters in adolescents aged 14 to 18 years with insomnia and intellectual disabilities in a placebo-controlled RCT, in which blood was taken every 15–60 min within 24 h via a venous vascular access to determine the melatonin concentration and polysomnographic controls were carried out [34].
- The first case report on the oral administration of melatonin during childhood and adolescence involved a blind child, aged 8, with non-24, for whom **an initial non-delayed oral dose of 0.5 mg was effective** (Figure 1) [39,40].
- In 74 children with insomnia in ASD in an open-label setting, Yuge et al. found that **1 mg of an orally administered rapid-release non-delayed melatonin preparation** was associated with a significant reduction in SOL (mean 37%; 95% CI 26 to 48%; *p* < 0.0001) [41].
- Van Geijlswijk et al. studied 72 children and adolescents with delayed sleep phase disorder (DSPD), who were treated in parallel in four groups for 1 week with 0.05, 0.1, or 0.15 mg/kg body weight (**1.6 mg**, 2.91 mg, or 4.39 mg, respectively) of melatonin or with a placebo. The results showed that **no dose–response correlations** were observed for these three doses, as several sleep parameters (sleep onset, SO; sleep onset latency, SOL; evening melatonin increase = dim light melatonin onset, DLMO) improved independently of the doses investigated (Figure 2) [42].

The effect on these parameters was stronger the earlier the oral melatonin was administered; significant correlations were observed between the time of evening melatonin administration, which took place in the evening between 5:58 p.m. and 8:17 p.m., and the shortening of sleep onset time or DLMO (*p* = 0.004 and 0.022, respectively) [42].

The authors suggested conducting further pharmacokinetic studies with lower doses to search for dose–response correlations.

The authors interpreted the surprising result as either of the following options:

(a) As a “lid effect” in relation to the saturation range of traditional dose–response curves, or (b) as an “all or nothing principle”, which was described in a comparable way [42].

- In 18 premature infants within their first week of life, the melatonin elimination half-life was between 16.9 and 21.0 h after intravenous melatonin administration [43]. In a further 15 premature infants, an elimination half-life of between 6.2 and 15.5 h was measured after intragastric melatonin administration via a nasogastric tube [44]. In nine children with ASD aged 3–8 years, the elimination half-life after the oral administration of 1 mg of melatonin was 1.3 ± 0.42 h (range: 0.68–2.0 h) [45]. Nine prepubertal adolescents had a slightly shorter elimination half-life than sixteen young adults (0.67 ± 0.12 vs. 0.79 ± 0.10 h, corresponding to a mean of 40.2 and 47.4 min, respectively) [46]. In young adults, the elimination half-life after the oral or intravenous administration of melatonin was less than one hour (53.7 + 7.0 min and 39.4 + 3.6 min, respectively) [15]. Other authors measured comparable values after the oral administration of melatonin in young adults aged 21 to 32 years (47 + 3 min) [47].
- Pharmacokinetic data on melatonin sustained-release preparations have not yet been published (except for the reference by Lalanne et al. in 2021 to unpublished data in an assessment report for the EMA from 2007 (https://www.ema.europa.eu/en/documents/scientific-discussion/circadin-epar-scientific-discussion_en.pdf (accessed on 9 May 2025), p. 16), which were measured by Ruth Kitzes-Cohen et al. in eight healthy male volunteers after the administration of a 2 mg sustained-release preparation for adults, and these would represent the only data to date on the pharmacokinetics of a melatonin sustained-release preparation with an active ingredient content of 2 mg [48]).

It can be concluded from these data that diagnosis- and age-related studies are required to clarify at which oral dose, in which formulation, and at what time melatonin can be recommended for children and adolescents with ASD and non-organic sleep disorders.

## 2. Method

**Inclusion criteria:** Randomised placebo-controlled trials (RCTs) in which the efficacy of treatment with melatonin in children and adolescents with ASD was tested (a) if ASD was specified as the sole diagnosis or (b) in which a subgroup analysis was performed in the presence of psychiatric comorbidities or other diagnoses.

**Exclusion criteria:** All studies that either did not address the topic of melatonin in ASD with non-organic sleep disorders in childhood and adolescence or that included ASD with psychiatric or other diagnoses without subgroup analysis.

**Figure 3:**
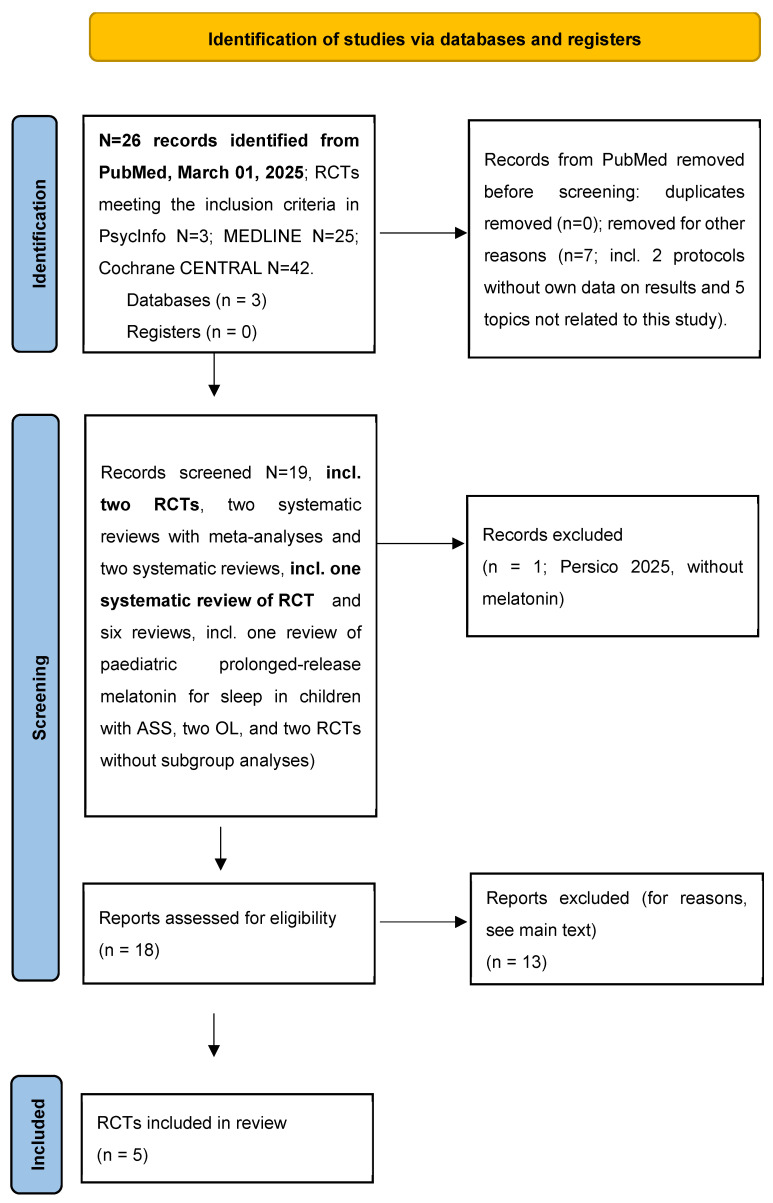
Flow chart showing the selection procedure applied here. For the list of excluded items, please see the Results and Supplementary S1: Protocols without own data on results [53,54] and 5 topics not related to this study [59,60,61,62,63]). Records screened N = 19, **incl. two RCTs** [7,49], two systematic reviews with meta-analyses [51,52], and two systematic reviews [50,55,56,57,58], **incl. one systematic review of RCT** [55] and six reviews [64,65,66,67,68,69], incl. one review of paediatric prolonged-release melatonin for sleep in children with ASS [67], two OL [70,71], and two RCTs without subgroup analyses [72,73]). Records excluded (without melatonin [50]). Source of the flow chart [74]. This work is licenced under CC BY 4.0.

Using the **PubMed** medical database on 1 March 2025, two authors (E.P.; A.S.), independently of each other, searched for the keyword combination “((melatonin[title/abstract]) AND (autism[title/abstract])) AND (randomised[Title/Abstract])”, taking all languages into account. In total, 26 publications were retrieved [7,49,50,51,52,53,54,55,56,57,58,59,60,61,62,63,64,65,66,67,68,69,70,71,72,73] (Figure 3). In these publications and in the literature references contained in these publications, we searched for RCTs on non-organic sleep disorders in children and adolescents with ASD. For studies that included not only ASD but also other underlying conditions such as SMS, fragile X syndrome, Rett syndrome, epilepsy, and/or ADHD, we checked whether subgroup analyses had been conducted for these diagnoses (see Supplementary S1).

**PsycINFO**, **MEDLINE**, and **Cochrane CENTRAL** were searched using the keyword combination “autism, melatonin, sleep”. In these databases, 3, 25, and 42 publications, respectively, were found, of which the same 4 RCTs fulfilled the inclusion criteria “childhood and adolescence” and “evidence of subgroup analyses in the presence of multiple initial diagnoses”, as in PubMed (see Figure 3 and Supplementary S1).

The searches were conducted independently by only two authors, as the number of studies found was limited to N = 26 (PubMed), and for each of these studies, it is declared in Supplementary S1 why it was included or excluded. In our view, a selection bias was thus ruled out (see Supplementary S2 and S3).

## 3. Results

### 3.1. The Results of the Five RCTs Representing the Outcome of the Present Systematic Review

At present, five randomised placebo-controlled studies on non-organic sleep disorders in children and adolescents with autism spectrum disorder (ASD) are available (Table 1), in four of which ASD was stated as the single underlying disease [6,7,75,76], and in the other study [49] (Table 1), ASD was declared with a differentiated evaluation in the form of subgroup analyses, with ADHD also being present in some of the patients. 

In an interesting study by Tse et al. in 2024 [6], it was unfortunately not recorded (a) at what time of day and in what lighting conditions the physical training took place and (b) whether the physical training was associated with changes in body weight or metabolic parameters. Further assessments of the quality of these RCTs are provided in Supplementary S2.

**Table 1:** **(a) Overview of the RCTs** available to date on the efficacy of melatonin in children and adolescents with non-organic sleep disorders and ASD alone or ASD in combination with other underlying conditions for which a subgroup analysis was presented. **(b) Characteristics of the RCTs** available to date on the efficacy of melatonin in children and adolescents with non-organic sleep disorders and ASD alone or ASD in combination with other underlying conditions for which a subgroup analysis was presented.

Author, Country	Diagnosis	N, Age	Melatonin (Dose, Preparation)	Result		Conclusion	
Garstang, 2006 [75] UK	ASS with insomnia.	**N = 7**, 5–15 years.	**Five-milligram oral** capsules, **immediate release,** 4 weeks vs. placebo, crossover after washout for 1 week.	**TST ↑**, **SOL ↓**, **WASO ↓**		This non-delayed preparation was effective in terms of falling asleep, sleeping through the night, and TST.	
Wright, 2011 [76] UK	ASS with insomnia.	**N = 17**, 9 ± 2.9 (4–16) years.	**Two-milligram oral non-delayed**, 30–40 min before the expected sleep; increased every 3 days by two milligrams **to max. ten milligrams (average seven milligrams)**.	**SOL ↓**, **TST ↑**, **WASO ↓ Improvement in behaviour, communication, and dyssomnia.**		This non-delayed preparation was effective in terms of falling asleep, sleeping through the night, TST, and improvement in behaviour and communication.	
Cortesi, 2012 [7] Italy	ASS with insomnia. *Comparison of melatonin alone and in combination with CBT (cognitive behavioural therapy; four sessions).*	**N = 134, ** 6.8 ± 0.9 years.	**Three milligrams of a combined preparation (one milligrams rapid release, two milligrams sustained release (6 h))** oral administration at 9:00 p.m. Four randomised groups in parallel; 3 months for each of the following: ● **Melatonin** (N = 34); ● **CBT** (N = 33); ● **Melat. + CBT** (N = 35); ● **Placebo** (N = 32).	**SOL ↓** to 44% with melatonin alone, to 23% after CBT, and to 61% with melatonin + CBT. **TST ↑**, **WASO ↓**, **sleep anxiety ↓** after KVT to 17%, after melatonin to 14%, and after both to 34%.		This combined 3 mg preparation alone was more effective than CBT alone. The combination of both was more effective than either method alone.	
Hayashi, 2022 [49] Japan	ASS with insomnia. *ADHD at 55% (108/196):* *comparable effects regarding improvement in SOL by melatonin in both doses.*	**N = 196**, aged 11.2 ± 2.5 (6–15) years.	Three parallel randomised, double-blind groups: **1 milligram or 4 milligrams non-delayed melatonin** vs. placebo for 2 weeks, administered 45 min before bedtime.	**SOL ↓, SE ↑**. Sleep hygiene alone in the prephase with lower effects compared to both melatonin doses.		The administration of this non-delayed preparation with 4 mg 45 min before bedtime was associated with comparable effects to 1 mg, but more frequent AEs. In the OL phase with up to 10 mg, a further increase in AEs was documented.	
Tse, 2024 [6] Hong Kong	ASS with insomnia	**N = 62**, aged 9.6 to 10.4 years	Four randomised arms: ● **Three milligrams of liquid Melatonin** 30 min before bedtime (N = 14); ● Cycling in the morning only (N = 18); ● Melatonin and cycling (N = 12); ● Placebo alone (N = 18).	**SOL** ↓, **TST** ↑, **SE** ↑, **WASO** ↑. Melatonin or cycling alone or melatonin with cycling exerted better effects compared to placebo.		The administration of this non-delayed liquid preparation with 3 mg 30 min before bedtime was associated with comparable effects to cyling. The combination of both methods was just as effective as each method on its own.	
**Author, Country**	**Diagnosis**	**N, Age, Dropouts, Missing Data**	**Target Values**	**Melatonin** **(Dose, Preparation)**	**Result**	**Conclusion**	**Side Effects**
Garstang, 2006 [75] UK	ASS with insomnia *.	**N = 7** (6 ♂), 5–15 years. * Dropouts: 36.4% (4/11). Missing data: none.	SOL; TST; WASO; waking-up activity in the morning via a sleep log written by parents.	**Five-milligram oral** capsules, immediate release ** 4 weeks vs. placebo, crossover after washout for 1 week. **Instructions on sleep hygiene** showed no effect before the start of the study and were maintained during the study.	Signif. ^#^ **TST ↑** 8.05 to 9.84 h; **SOL ↓** 2.60 to 1.06 h; a**wakenings ↓** from 0.35 to 0.08.	**This non-delayed preparation was effective in terms of falling asleep, sleeping through the night, and TST.**	No data available.
Wright, 2011 [76] UK	ASS with insomnia ***.	**N = 17** (10 ♂), 9 ± 2.9 (4–16) years. Dropouts: 15% (3/20). Missing data: 5.9% (1/17). *Previous statistical case number planning and power analysis.*	As above; also, four standardised questionnaires on sleep, behaviour, health, and complications ^##^.	**Two-milligram oral non-delayed** ^§^, 30–40 min before the expected sleep; increased by the parents every 3 days by two milligrams **to max. ten milligrams (average seven milligrams)** until “good sleep” with an “improvement of ≥50%” was achieved. **Instructions on sleep hygiene** showed no effect before the start of the study and were maintained during the study after further instruction of the parents.	**SOL ↓** 135 to 82 min (baseline vs. melatonin at bedtime), 130 vs. 78 min on melatonin application vs. placebo. **TST ↑** 500 to 556 min. Number of a**wakenings ↓** 0.5 to 0.43/night. **Improvement in behaviour, communication, and dyssomnia.**	**This non-delayed preparation was effective in terms of falling asleep, sleeping through the night, TST, and improvement in behaviour and communication.**	No statistically significant differences between verum and placebo with regard to all characteristics such as headache, vomiting, reduced appetite, and reduced attention, according to SEQ ^##^.
Cortesi, 2012 [7] Italy	ASS with insomnia ^+^. *Comparison of melatonin alone and in combination with CBT (cognitive behavioural therapy; four sessions).*	**N = 134 ** (82% ♂ in the melatonin group with a total of 34 children) aged 6.8 ± 0.9 years. Dropouts: 10% (16/160). Missing data: 6.9% (10/144).	SOL; TST; WASO (sleep diary and actigraphy); two standardised questionnaires on sleep and behaviour ^++^.	**Three milligrams of a combined preparation (one milligram rapid release, two milligrams sustained release (6 h))** ^§§^ oral administration at 9:00 p.m. Four randomised groups in parallel; 3 months each of following: ● **Melatonin** (N = 34); ● **CBT** (N = 33); ● **Melat. + CBT** (N = 35); ● **Placebo** (N = 32).	**SOL ↓** to 44% with melatonin alone, to 23% after CBT, and to 61% with melatonin + CBT. **TST ↑. Number of awakenings ↓.** **Sleep anxiety ↓** after KVT to 17%, after Melat. to 14%, and after both to 34%.	**This ** **combined 3 mg preparation alone was more effective than CBT alone. The combination of both was more effective than either method alone.**	No side effects were observed.
Hayashi, 2022 [49] Japan	ASS with insomnia. ADHD at 55% (108/196): comparable effects regarding improvement in SOL by melatonin in both doses. No differences regarding the improvement in SOL after 1 mg or 4 mg of melatonin in children with a height <145 cm.	**N = 196, ** (62% ♂) aged 11.2 ± 2.5 (6–15) years. Dropouts: 14.4% (33/229). Missing data: 4.6% (9/196). *Preliminary statistical case number planning and* *power analysis.*	SOL; TST; SE; WASO; sleep diary; actigraphy; standardised recording of five characteristics for irregular behaviour (ABC-J); height; weight; standardised checklist for recording adverse events (AEs)) ^+++^.	Three parallel randomised, double-blind groups: **1 mg or 4 mg non-delayed melatonin** vs. placebo for 2 weeks, administered 45 min before bedtime. Then, 42 days OL with dose increase if required after 7 days, up to max. 4 mg. Then, 14-day follow-up to exclude rebounds and withdrawal symptoms. ***Compliance with defined instructions on sleep hygiene before and during the study.***	**SOL ↓** to 21/20/1 min after 1 mg mel./4 mg mel./placebo, respectively (actigraphy, *p* < 0.0001). **SE ↑/=** to 2.35 resp. 2.07% after 4 mg resp. 1 mg (*p* = 0.04 resp. 0.13 n.s.). TST unchanged. WASO unchanged. **Sleep hygiene alone in the prephase with lower effects compared to both MEL doses.**	**The administration of this non-delayed preparation with 4 mg 45 min before bedtime was associated with comparable effects to 1 mg, but more frequent AEs.** **In the OL phase with up to 10 mg, a further increase in AEs was documented.**	Discontinuation at 4 mg during the RCT phase in one child due to AEs. No change in the five characteristics for aberrant behaviours in the remaining children during the RCT phase according to parents and physicians. Drug-related AEs 0/5/3 = 0%/7.7%/4.5% for 1 mg/4 mg/placebo during RCT. AEs total incl. the OL phase: RCT 1 mg: 13.8%; RCT 4 mg: 29.2%; RCT Plac.: 18.2%; OL 1–10 mg: 36.3%.
Tse 2024 [6] Hong Kong	ASS with insomnia	**N = 62** (81% ♂; nine boys and five girls in the melatonin group) aged 9.61 to 10.36 years. Dropouts: 22.5% (18/80). Missing data: none. *Preliminary statistical case number planning and* *power analysis.*	SOL; TST; SE; WASO; sleep diary; actigraphy. ^$^	Four randomised arms for 2 weeks: **3 mg liquid melatonin** 30 min before bedtime (N = 14); cycling 5 × 60 min/week (N = 18); melatonin and cycling (N = 12); placebo (N = 18).	**SOL** ↓, **TST** ↑, **SE** ↑**, WASO** ↑. Melatonin or cycling alone or Melatonin with cycling with better effects compared to placebo (Figure 4).	**The administration of this non-delayed liquid preparation with 3 mg 30 min before bedtime** was associated with comparable effects to cycling. The combination of both methods was just as effective as each method on its own (Figure 4).	No side effects were observed after the administration of melatonin.

*: No ADHD as concomitant disorder; initial group N = 11; evaluation of seven cases with complete data sets; in the initial group, two had dyspraxia, one had severe learning difficulties, two had moderate and three had mild learning difficulties. ASD was confirmed using four cumulative criteria, as follows: (a) difficulties in verbal and non-verbal communication, (b) difficulties in social interaction, (c) perceptual disorders (“lack of imagination”), and (d) diagnosis confirmed by a paediatric and adolescent psychiatrist. Insomnia was defined using five criteria, as follows: (a) sleep latency ≥ 1 h or nocturnal waking with parental stress, (b) at least 4 nights/week, (c) persistent for at least 6 months, (d) with effects on the child or family members, and (e) lack of effect of behavioural management techniques. **: Company Penn Pharmaceuticals Ltd., Tridig UK. **#:** No indication of the statistical method; indication of mean values, 95% confidence intervals, tables and graphs for all results. ***: Diagnosis of ASD according to ICD 10 criteria; if uncertain, additional standardised tests were used (ADOS and ADI-R). Insomnia is defined as “sleep disorders” including increased sleep latency, insomnia, “excessive nocturnal waking”, or reduced TST. ^##^: Questionnaires: SDQ = Sleep Difficulties Questionnaire, DBC = Behavioural, five subscales on anxiety, disruptive behaviour, etc., GHQ = General Health Questionnaire, and SEQ = Side Effects Questionnaire. **^§^:** Company “DHP pharma”, UK. **+:** Insomnia: Within 3 months prior to screening for the study and during the 14-day screening phase, on at least 3 nights per week, SL > 20–30 min, and waking after falling asleep for > 30 min; ASA according to DSM IV-TR, incl. ADOS and ADI-R testing; exclusion of brain malformations, epilepsy, metabolic diseases, chromosomal abnormalities, and perceptual disorders by means of MRI, EEG, evoked potentials, high-resolution karyotyping, and neurometabolic laboratory screening. Preliminary exclusion of obesity, sleep-related breathing disorders (anamnestic), and exclusion of periodic leg movements during sleep during the 12-week study. **++:** CBCL = Child Behaviour Checklist, CSHQ = Children’s Sleep Habits Questionnaire with 33 questions. §§: Armonia Retard 3 mg, Nathura, Montecchio Emilia, Italy. **+++:** ASD diagnosis, according to DSM V; sleep disturbance defined via SL ≥ 30 min on at least 3 days/week in the last 3 months, measured using an electronic diary; actigraphy using an FS-760 device (only movement analyses), standardised checklist for recording NW according to Aman 1994 (AEs, documented in detail in the Supplementary Materials); structured, consistent instructions on sleep hygiene before and during the study, no concomitant medication, exclusion of patients with indications of sleep-related breathing disorders, severe intellectual impairment, liver dysfunction, bipolar disorders, and/or schizophrenia. Sponsor and provision of the MEL preparation and the placebo: Nobelpharma C., Ltd. $: Confirmation of the diagnosis of ASD according to DSM-5 including Autism Diagnostic Interview-Revised (ADI-R) in prepubertal children and adolescents (Tanner stage I), SOL ≥ 30 min according to parents >3×/week, non-verbal IQ ≥ 50 (WISC, Chinese revised), Social Response T-Score ≥ 75, ability to ride a two-wheel bicycle, no drug treatment in the last 4 weeks before study start and no previous treatment with melatonin; exclusion of psychiatric comorbidities, physical limitations and neurological diseases such as epilepsy, fragile X syndrome and tuberous sclerosis as well as exclusion of previous training effects, e.g., in the form of 60 min of physical training per day. Actigraphy and sleep diary to record the above-mentioned parameters. Melatonin liquid, Natrol^®^ Chatsworth, CA, USA.

### 3.2. Critical Comparison with the Results of Reviews by Other Authors

In the reviews by Zisapel (2022) [65] and Schröder et al. (2021), cited in Zisapel (2022) [77] as well as Cortese et al. (2020) [66], Parker et al. (2019) [51] and Rossignol and Frye (2011) [52], no further RCTs are specified that fulfil this requirement. In the study by Schröder et al. (2021), children with ASD and Smith–Magenis syndrome were treated with a prolonged-release preparation; no subgroup analysis was performed [67]. In another systematic review of pharmacological treatments for sleep disorders in children, McDonagh et al. (2019) found 19 RCTs on melatonin [56], including 4 RCTs on ASD, which are included in Table 1 [75,76] or in the discussion of this systematic review [72,73]. Maras et al. (2018) [70] investigated a delayed melatonin preparation in children with ASD using the OL method, but not in a randomised setting.

In the detailed review by Tordjman et al., 2013, with 161 references, seven RCTs on melatonin in ASD are listed, which are also considered in the current systematic review [69]. In addition, Tordjman et al. listed three case reports, three retrospective studies, and six OL studies on melatonin in ASD, as well as seven studies in which melatonin concentrations in blood or urine were analysed in ASD patients in their table. These included only one study on serum melatonin concentrations in fourteen children with ASD, four of which showed an inverted day–night profile, as in SMS (Kuhlman et al., 2000) [78].

### 3.3. Missing Subgroup Analyses in RCTs on Patients with Multiple Diagnoses

Further studies also investigated melatonin in ASD; however, no conclusions specific to the diagnosis of ASD can be drawn from these studies, as no subgroup analyses were carried out, although one or more additional diagnoses were stated at the same time, as follows:

- Gringras et al. reported on 121 children and adolescents with **ASD** and 4 children and adolescents with **Smith–Magenis syndrome** without differentiating the subgroups, in which 28.8% of the patients participating in the randomisation had **ADHD** and 12.8% had **epilepsy** [72]. Only 46.8% (125/267) of the originally recruited patients were included in the randomisation. With dropout rates of 15% (9/60) and 32.3% (21/65) in the placebo group, data from *51 verum and 44 placebo “cases”* were finally analysed. This study of the sustained-release formulation was financed by the manufacturer and statistically analysed under its responsibility.
- Schröder et al. published an analysis of some of the above data in 2019 [67]. Malow et al. analysed the data of the same(?) 51 verum vs. 44 placebo “cases” again in 2021, again without differentiating between the above-mentioned subgroups [79]. In 2012, Malow et al. had previously reported on different doses of melatonin in 24 children and adolescents with difficulty falling asleep and ASD, but without a randomised setting [71].

### 3.4. Differences in Circadian Rhythmicity in Patients with ASD or Smith–Magenis Syndrome

**ASD and Smith–Magenis syndrome (SMS) are fundamentally different clinical pictures in terms of their circadian melatonin rhythms and cannot be compared with each other, as discussed below:**

- In patients with SMS, no or very low melatonin concentrations are spontaneously detectable in the saliva between 23:00 and 07:00 without prior melatonin input, while, during the day, the paradoxical melatonin releases typical of SMS are detectable [80].
- ASD, on the other hand, is associated with endogenous melatonin release at night between 22:00 and 04:00 [45].
- The joint biostatistical evaluation of the circadian rhythm of peripheral melatonin concentrations in the blood, saliva, or urine of patients with ASD and SMS can therefore lead to the assumption of a nocturnal melatonin deficiency in ASD which, however, would only have to be analysed for this subgroup alone in order to obtain comprehensible conclusions.
- The genetic background for chronodisruption with a reversal of the day–night phase in patients with SMS is, in most cases, a 17q.11.2 microdeletion, which is associated with a functional disorder of the retinoid acid-induced 1 gene (RAI1) [81,82].
- There was a reciprocal difference between patients with ASD and SMS in terms of the frequency of disorders of social communication behaviour (4:1 vs. 1:3) [83].

- Wirojanan et al. also reported on a small group of patients in which no subgroup analyses were performed (five patients with ASD alone, three patients with ASD with fragile X syndrome, and four patients with fragile X syndrome) [73].
- Wasdell et al. analysed the data of 16 patients with ASD without subgroup analysis, as part of an unspecified group of 47 patients with neurodevelopmental disorders [84].

### 3.5. Pharmacokinetics of Melatonin in Children and Adolescents with ASD

To our knowledge, dose–response correlations between melatonin and sleep in children and adolescents with ASD, with the documentation of melatonin concentrations in the blood, have only been investigated by Goldman et al., in their observational study involving nine children [45]. After the oral administration of a rapid-release, non-delayed melatonin preparation, significantly different melatonin concentrations in the blood were found (Figure 5) [45]. In all nine children, sleep onset latency was associated with a rapid initial increase in plasma melatonin concentration (Figure 5). The mean sleep duration after the administration of 1 mg of melatonin tended to be higher (480 min) than after the administration of 3 mg of melatonin (380 min); this difference was not significant in view of the small sample size (see Figure 5 for further details).

In their systematic review [85], Carmassi et al., 2019, referred to two further studies in which melatonin concentrations in the blood were measured, which investigated 10 adults with ASD (Nir et al., 1995) [86] and 14 prepubertal children with ASD aged 5–10 years (median 7 years) (Kulman et al., 2000) [78]. Unfortunately, no individual measurements were given in the Kulman study, so no pharmacokinetic parameters can be derived from these data. In addition, higher melatonin concentrations during the day than at night were measured in four of the ten children; therefore, from our point of view, it cannot be ruled out that these four patients were children with SMS and ASD. The reported mean values of the serum concentrations of this study, therefore, cannot be analysed pharmacokinetically.

Tordjman et al. showed that, in 50 children with ASD with a mean age of 11.5 + 4.5 years, compared to 88 healthy controls, the nocturnal excretion of the melatonin metabolite 6-sulfatoxymelatonin was only reduced prepubertally, while, pubertally and postpubertally, there were no differences [87]. However, this study did not take into account that the concentration of 6-sulfatoxymelatonin in urine is influenced by the urine flow rate, so the measured concentrations in urine are related to the creatinine concentration [88]. Using this procedure, no significant correlations between the corrected 6-sulphatoxymelatonin concentrations in urine and sleep parameters (measured with the standardised CSHQ Sleep score) were detectable in 56 children and adolescents with ASD aged 5.3 + 2.4 years (range: 2.8–13.3 years) [89].

## 4. Discussion

### 4.1. Importance of Diagnosis-Related RCTs with Subgroup Analyses

To date, five randomised placebo-controlled trials (RCTs) have been conducted in which the efficacy of orally administered melatonin on non-organic sleep disorders in children and adolescents, either with the sole underlying diagnosis of autism spectrum disorder (ASD) or with additional diagnoses, for whom subgroup analyses were conducted, was tested (Table 1). In these studies, non-delayed preparations that release the active substance rapidly were used (Garstang 2006, Wright 2011, Cortesi 2012, Hayashi 2022, Tse 2024) [6,7,49,75,76]; in one of these studies, a mixed preparation with a non-delayed preparation of 1 mg and a delayed portion of 2 mg was applied (Cortesi 2012) [7].

To our knowledge, dose–response correlations of melatonin and sleep in children and adolescents with ASD have only been investigated by Goldman et al. in an observational study involving nine children [45]. After the oral administration of a rapid-release, non-delayed melatonin preparation of 1 mg or 3 mg, there were no dose–response correlations and significantly different melatonin concentrations in the blood; without exception, the sleep onset latency was associated with a rapid initial increase in melatonin concentrations in the evening [45].

Similarly, van Geijlswijk et al. also found no dose–response correlations after the administration of a non-delayed melatonin preparation in paediatric and adolescent patients with delayed sleep onset (DSPD) at doses of 0.5, 1.0, or 1.5 mg of melatonin/kg body weight [42]. To date, no studies have been published on the efficacy and pharmacokinetics of delayed melatonin preparations in paediatric, adolescent, and adult patients with a single initial diagnosis of ASD or with ASD in combination with several underlying diagnoses such as ADHD, epilepsy, Smith–Magenis syndrome, and/or fragile X syndrome and corresponding subgroup analysis. In an EMA assessment report from 2007, only brief summary data were given with regard to pharmacokinetic data following the administration of a 2 mg delayed preparation to eight adults aged 55 years and older without any indication of ASD (see p. 16 in the following document: https://www.ema.europa.eu/en/documents/scientific-discussion/circadin-epar-scientific-discussion_en.pdf (accessed on 17 March 2025)).

De Souza et al. showed that children and adolescents with ADHD have reduced pineal melatonin synthesis and secretion at night [90]. This observation supports the need for the effects of melatonin to be considered in relation to the initial diagnosis (the underlying disease), on the basis of which a non-organic sleep disorder can be observed and treated with melatonin.

### 4.2. Quality Heterogeneity of Existing RCTs with Regard to the Use of Adequate Parameters and Methods for the Assessment of Sleep and the Different Types of Sleep Disorders

The quality of the RCTs available to date on the effectiveness of melatonin in non-organic sleep disorders associated with neurogenetic diseases including ASD is heterogeneous; in particular, there is a lack of standardised and easily comparable parameters that investigate sleep and sleep quality and their effects on well-being and performance during the day [35]. Within the five RCTs listed in Table 1, we can see that the quality and differentiation of the studies have improved over time. Cortesi et al. showed that cognitive behavioural therapy can also be effective in children and adolescents with ASD, with melatonin having more pronounced effects [7]. However, the combined use of CBT and melatonin showed the highest efficacy. Hayashi et al. showed that subgroup analyses can increase the value of an RCT [49].

### 4.3. Physiological and Pathophysiological Basics

**Figure 4:**
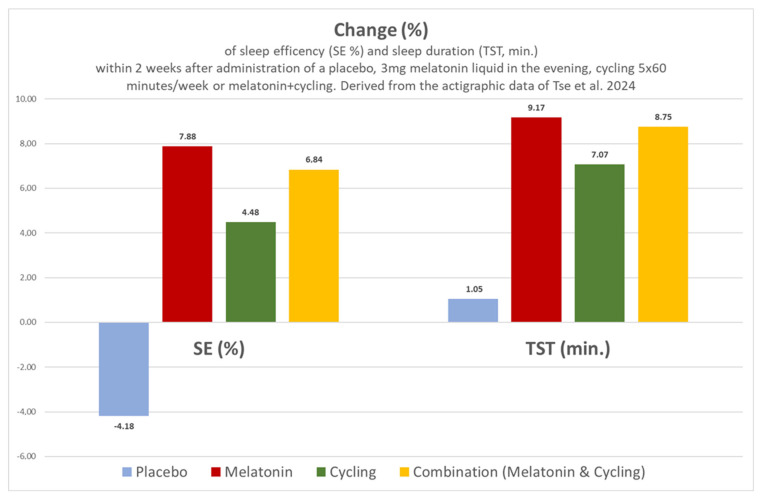
Change in sleep efficiency (SE %) and sleep duration (TST, min) within 2 weeks after administration of placebo, 3 mg of melatonin liquid in the evening, cycling 5 × 60 min/week or melatonin + cycling. Derived from the mean values of the actigraphic data from Tse et al. 2024 [6]. The duration of nocturnal awakening (WASO, min) increased by 21.5% after two weeks of placebo administration, while the duration of nocturnal awakening decreased by 26.90%, 18.61% and 26.30% after the administration of melatonin, cycling, and the combined intervention of melatonin + cycling, respectively.

**Figure 5:**
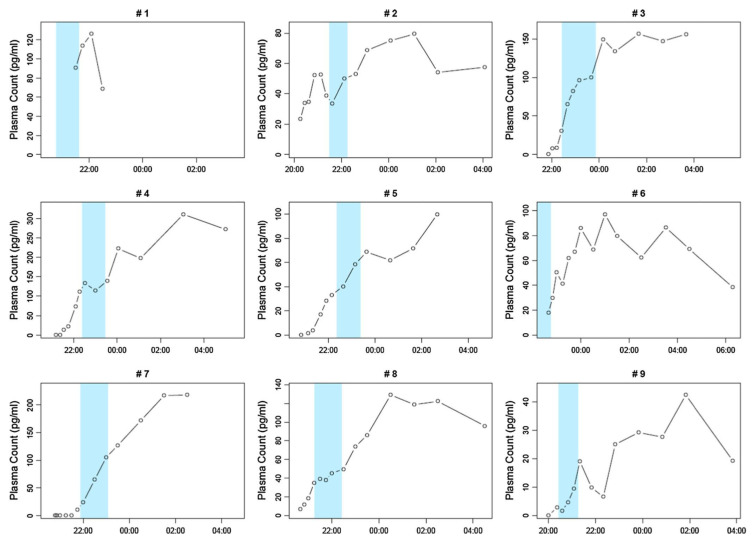
Plasma melatonin concentrations in 9 children with autism spectrum disorder, aged 3–8 years, in relation to sleep onset latency (SOL; blue columns; Goldman et al. 2014) [45]. The blue columns show when each child was placed in bed and how long it takes for the child to fall asleep (= SOL). This time point and the associated duration were plotted in 9 children with autism spectrum disorder in relation to the course of the melatonin concentrations in the serum. In all 9 children, falling asleep was associated with a rapid initial increase in melatonin concentration in the evening. The polysomnographically determined sleep duration (TST, total sleep time) did not improve significantly after the evening administration of 1 mg or 3 mg of melatonin (*p* = 0.35); a longer mean sleep duration of 468 min was more likely to be observed with the lower dose of 1 mg than after the administration of 3 mg of melatonin (initial value: 476 + 72.2 min; after the administration of 1 mg of melatonin: 468 + 153.8 min; after 3 mg: 380.4 + 187.8 min; [45]). Reproduced with permission.

**Figure 6:**
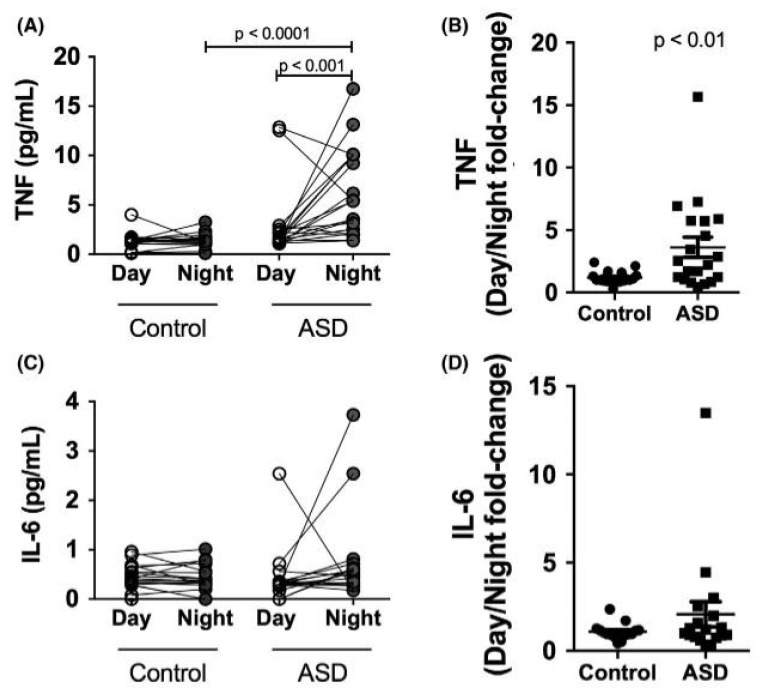
(**A**) TNF and (**C**) Interleukin-6 concentration in saliva of 20 children with autism and sleep disorders vs. 20 healthy controls aged 4–18 years during the day and at night; (**D**) and (**B**) Day/Night fold-change of the same parameters; data are from Sanseray da Silveira Cruz-Machado et al., 2021: **Disrupted nocturnal melatonin in autism**: Association with tumour necrosis factor and Interleukin-6 with sleep disturbances [104]; a review by this group of authors on this topic can be found in Pinato et al. 2019 [105]. Reproduced with permission.

To understand the use of melatonin in children and adolescents with ASD, some recent data on the pathophysiology of the pineal gland and the specificities of the effects of melatonin in ASD are recalled, as follows:

(a) The pineal gland appears to be involved in the pathogenesis of autism spectrum disorder (ASD) in the following two ways:
  - Based on the retrospective comparison of numerous data from children with ASD compared to control groups, Miike et al. (2020) assumed that the development of chronobiological rhythms in children with ASD can be disturbed by the following factors: maternal bed rest only after midnight, prematurity, irritability, and a tendency towards disturbed sleep in early infancy [91].
  - Shomrat and Nesher (2019) summarised the results of numerous studies on detailed questions of ASD pathogenesis [92]. As endogenous N,N-dimethyltryptamine (DMT) is formed and secreted in the pineal gland, in addition to the pulsatile circadian nocturnal synthesis and secretion of melatonin, synaptogenesis and neuroplasticity could be disturbed via altered DMT activity and the reduced melatonin concentrations in ASD, thereby resulting in the development of ASD [92]. DMT is also formed from tryptophan and classified as a neurotransmitter [93].
(b) Hayashi et al. (2022) identified seven further pathogenetic factors in relation to melatonin in ASD, as follows: abnormalities in synthesis, concentration, secretion patterns, metabolism (such as polymorphisms of genes involved in the formation of pineal enzymes for the synthesis of melatonin = polymorphisms of the AOMT gene = the acetylserotonin-O-methyltransferase gene); impaired signalling to melatonin 1A receptors; the dysregulation of immunological signalling; and the inflammation of the central and peripheral immune system (Hayashi 2022) [49]. These factors have also been considered in several whole-genome association studies and comparable studies in which associations with such pathways have been demonstrated [94,95,96,97,98].
(c) The “disruption of nocturnal melatonin synthesis and secretion” observed in children with autism is associated with measurable interleukin-6 and tumour necrosis factor activations during sleep, which are detectable in ASD but not in healthy controls (Figure 6); for a recent review of these neuroimmunological features in ASD, see Hughes et al. (2023) [99]. To our knowledge, clinical studies have not yet investigated whether the anti-inflammatory and immunomodulatory effect of melatonin [100,101,102] is of clinical significance in children and adolescents with ASD.
(d) Goldman et al. (2014) showed that, in pharmacokinetic studies on endogenous melatonin concentrations in the blood of children with ASD, in therapeutic terms, it is not a question of replacing reduced melatonin concentrations, as there are no simple dose–response relationships. After the oral administration of 1 mg of melatonin, the measured melatonin concentrations were significantly higher than endogenous melatonin concentrations: “suggest[ing] that supplemental melatonin is not replacing a deficiency state but has other mechanisms of action” (Figure 4; Goldman et al. 2014, p. 9) [45].

Similarly, Claustrat (2015) pointed out that, with regard to melatonin, it is the dose and not the duration of the hormonal signal that is decisive [103].

**Figure 7:**
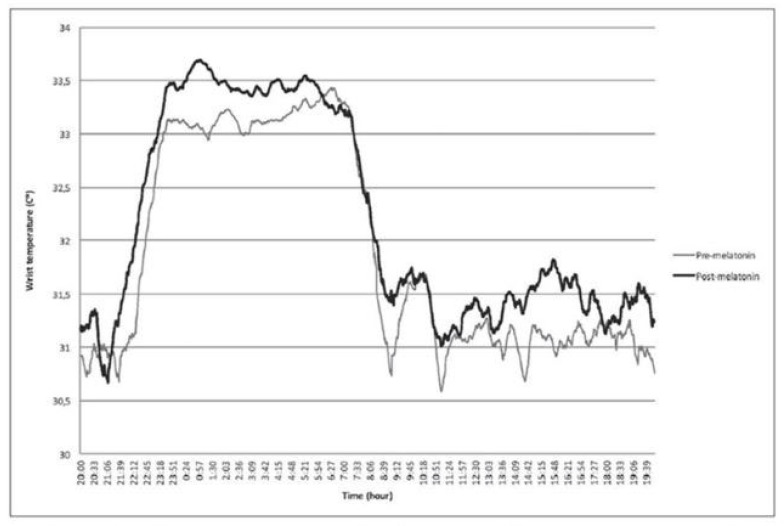
Increase in actigraphically measured peripheral temperature as a marker for reduction in core body temperature due to 2 mg of immediate-release melatonin administered orally 30 min before bedtime, effective prospectively over an observation period of 2 months; N = 26 children with autism spectrum disorder aged 6–18 years. This is parallel to a decrease in sleep latency from 22.6 (14.5–43.0) minutes to 9.5 (5.8–28.6) minutes (*p* < 0.01) and increase in sleep efficiency from 86.5 to 89.4% (*p* < 0.05). From Martinez-Cayuelas E et al. 2023 [106]. Reproduced with permission.

Martinez-Cayuelas E. et al. (2023) reported on the effect of melatonin on sleep in children with ASD as a result of the first published actigraphic temperature, light, and movement analyses in 26 children with ASD aged 6–18 years. The administration of a non-delayed immediate-release melatonin preparation (2 mg, orally, 30 min before bedtime) led to an immediate and sustained increase in peripheral temperature as a marker for lowering core temperature for several hours. This temperature effect is mediated via melatonin receptors type 2 [106] and was demonstrable in this prospective study, as was the improvement in sleep latency and sleep efficiency within the observation period of 2 months (Figure 7) [106].

### 4.4. Strengths and Limitations of the Present Systematic Review

In our view, this systematic review has the following strengths and limitations:

**Strengths:** We included the expertise of paediatricians (E.P., B.S., O.I.) and a psychologist (A.S.) who have been clinically and scientifically involved with sleep disorders in childhood and adolescence for several years; in addition, the expertise of a clinically and scientifically experienced pharmacologist (B.R.) and biostatistician (R.K.) was included. The searches were carried out in the four databases PubMed, MEDLINE, PsycInfo and Cochrane CENTRAL. All languages were taken into account. The inclusion and exclusion criteria were rigorously and objectively defined and transparently described (see Supplementary S1).

With the RCTs by Cortesi et al. 2012 [7] and Tse et al. 2024 [6], two studies are available in which the effect of two interventions on the sleep of children and adolescents with insomnia in ASD was tested in a randomised setting (melatonin alone vs. placebo or melatonin AND cognitive behavioural therapy or exercise). In both studies, it was ensured that there was no concomitant medication and that there was no comorbidity such as ADHD, SMS or tuberous sclerosis. Subgroup analyses were, therefore, not necessary. Almirall et al. published interesting biostatistical information on how similar RCTs can also be organised for sequential interventions [107].

**Limitations:** The database searches presented here were conducted by only two authors (E.P., A.S.). In our view, selection bias was largely ruled out, as the number of studies found in four databases was limited and as the reasons for inclusion or exclusion based on the pre-declared inclusion and exclusion criteria were explained transparently for each study in Supplementary S1. A meta-analysis of the five included RCTs was not conducted, as the number of available RCTs appeared to be too small. As the RCTs listed in Table 1 only examined rapid-release oral preparations of melatonin, no statement can be made about the efficacy of oral prolonged-release preparations. In addition, to the best of our knowledge, there are no RCTs with subgroup analyses to date in which the efficacy of sustained-release and non-released melatonin preparations is available. The number of studies on the pharmacokinetics of melatonin in childhood and adolescence is insufficient, meaning that there is only a limited database on changes from infancy to adolescence.

In our view, the understanding of the pharmacokinetics of melatonin in childhood and adolescence could be significantly improved if ethically acceptable ways were sought to investigate the relationships between melatonin in CSF and blood in children and adolescents with ASD, ADHD and Smith–Magenis syndrome. This could be considered in individual cases (a) if a CSF drainage is already in place, e.g., in the case of hydrocephalus, (b) if a neurosurgical operation or lumbar puncture is required anyway, during which a small amount of CSF could be taken for analysis, or (c) if an autopsy is required. Such investigations have been presented in a comparable manner in patients with hydrocephalus [108,109]—also in children with hydrocephalus [108]—or in animal experiments [25,26,110,111,112,113,114,115,116]; see also the reviews in [117,118,119]. These data indicate that the pineal gland as a circumventricular organ secretes the circadian melatonin produced in the pinealocytes during external darkness with a rapid and high increase in concentration into the ventricular cerebrospinal fluid. In the peripheral blood circulation, pineal melatonin appears delayed with a considerably lower concentration than in the cerebrospinal fluid. The melatonin concentration in the blood, therefore, has only limited diagnostic value. Debus et al. showed in a child with hydrocephalus that after oral administration of 5 mg of melatonin, a rapid increase in the melatonin concentration in the cerebrospinal fluid occurred within 10 min; the child fell asleep 40 min after melatonin administration. Within the following 7 h, the melatonin concentration decreased exponentially [108]. These data support the assumption that falling asleep in the dark is physiologically associated with a rapid increase in the melatonin concentration in the cerebrospinal fluid, an argument in favour of the oral administration of rapid-release, non-delayed melatonin preparations.

## 5. Conclusions

To our knowledge, there are no RCTs to date in which the efficacy of sustained-release melatonin preparations has been tested in children and adolescents with ASD and non-organic sleep disorders.

To our knowledge, five RCTs on ASD have been conducted to date in which non-delayed rapid-release preparations were tested and showed efficacy in relation to sleep parameters (Table 1). In one of these studies, a combination preparation with rapid-release melatonin and a sustained-release component was tested; in this study, it was shown in one study arm that using cognitive behavioural therapy in conjunction with this melatonin preparation was more effective than either therapy alone. This result is remarkable, as it demonstrates that cognitive behavioural therapy can also be effective in children and adolescents with ASD and non-organic sleep disorders.

No subgroup analyses were carried out in other RCTs on the efficacy of melatonin in ASD, so no conclusions can be drawn from these studies on the diagnoses of these subgroups, which were only analysed in a mix, such as ASD, Smith–Magenis syndrome, epilepsy, and so on, that would allow conclusions to be drawn about the efficacy of delayed melatonin preparations in ASD.

To date, there have been no RCTs carried out in which the efficacy of rapid-release melatonin preparations has been investigated in comparison to sustained-release melatonin preparations in children and adolescents with non-organic sleep disorders. Physiological and pharmacokinetic data available to date indicate that there are no classic dose–response relationships and that rapid-onset (i.e., rapid-release) preparations are effective even at low doses (1 mg in the evening in adults [120]; 0.25 to 0.5 mg in children and adolescents [11,120]). It should be taken into account that the elimination half-life of melatonin in infancy is much longer than in young adulthood; therefore, a longer elimination half-life can also be assumed in childhood and adolescence.

The studies cited in Table 1 and the physiological and pathophysiological principles mentioned in the discussion indicate that, in addition to psychosocial factors, the evolutionary biological relationships between melatonin, sleep, circadian chronobiological rhythms, immunology, genetics, epigenetics, motor skills and physical activity, temperature regulation and behaviour should also be taken into account in children and adolescents with ASD.

## Supplementary Materials

The following supporting information can be downloaded at: https://www.mdpi.com/article/10.3390/children12050648/s1.

## Abbreviations

AE	Adverse event
AOMT	Acetylserotonin-O-methyltransferase
ASD	Autism spectrum disorder
CBT	Cognitive behavioural therapy
CSF	Cerebrospinal Fluid
DLMO	Dim light melatonin onset
DMT	N,N-dimethyltryptamine
DSPD	Delayed sleep phase disorder
EMA	European Medicines Agency
G-BA	Joint Federal Committee (Gemeinsamer Bundesausschuss)
Non24	Non-24 syndrome; deviation from 24 h circadian rhythmicity
OL	Off-label
RCT	Randomised controlled trial
SE	Sleep efficiency
SMS	Smith–Magenis syndrome
SO	Sleep onset
SOL	Sleep onset latency
TST	Total sleep time
WASO	Wake after sleep onset = awakenings; number and duration
