# Supplementary material for: The Pharmacokinetics, Dosage, Preparation Forms, and Efficacy of Orally Administered Melatonin for Non-Organic Sleep Disorders in Autism Spectrum Disorder During Childhood and Adolescence: A Systematic Review
Source: Children (Basel). 2025 May 16;12(5):648. doi: 10.3390/children12050648 (PMC12110111; doi:10.3390/children12050648)
Supplement: Supplementary file 1 [file children-12-00648-s001.zip › Suplement 1_Paditz E et al.pdf]

Children 2025, Autism/Melatonin (Paditz, Renner, Koch, Schlarb, Schneider, Ipsiroglu)

Supplement 1 Reasons why studies from the PubMed, MEDLINE, PsycInfo and Cochrane CENTRAL databases were included or excluded. 'References'= RCTs that meet the inclusion criteria (in the bibliographies of the articles found by the search). incl. = included, excl. = excluded articles.

No.	Author, Year	Pub Med N=26	MED LINE N=25	Psych INFO N=3	Coch-rane CEN-TRAL N=42	References	incl.	excl.	comment
**PubMed**									
1	Persico 2025 [1]	X						X	1)
2	Liang 2024 [2]	X						X	melatonin Ø
3	Raghavan 2023 [3]	X						X	melatonin Ø
4	Tse 2022 [4]	X						X	protocol
5	Schröder 2022 [5]	X						X	review 2)
**6**	**Hayashi 2022 [6]**	**X**			**X**		**X**		**RCT with subgroup analyses, see Tab. 1**
7	Zisapel 2022 [7]	X						X	review 3)
8	Ayuse 2020	X						X	melatonin Ø
9	Parvatanen i 2020 [8]	X						X	review 4)
10	Cortese 2020 [9]	X						X	review 5)
11	Schroder 2019 [10]	X						X	RCT without subgroup analysis
12	Parker 2019 [11]	X				Garstang 2006 [12]; Wright 2011 [13]; Cortesi 2012 [14]		X	systematic review and meta-analyses: oral melatonin in children with neurodisabilities 6)
13	Goltz 2019 [15]	X						X	review 7)
14	McDonagh 2019 [16]	X				Wright et al. 2011 [13]; Garstang et al. 2006 [12]		X	systematic review 8)
15	Maras 2018 [17]	X						X	´A prospective, **open-label** efficacy and safety follow-up of nightly ... melatonin´

16	Gringras 2017 [18]	X						X	RCT **without subgroup analyses**
17	Klein 2016 [19]	X						X	review 9)
18	Rossignol 2014 [20]	X				Garstang 2006 [12]		X	review
19	Tordjman 2013 [21]	X				Garstang 2006 [12] Wright 2011 [13], Cortesi 2012 [14]		X	review
**20**	**Cortesi 2012 [14]**	**X**					**X**		**RCT, see Tab. 1**
21	Malow 2012 [22]	X						X	´an **open-label** dose-escalation study´
22	Anagnostou 2011 [23]	X				Garstang 2006 [12]		X	review
23	Guénolé 2011	X				Garstang 2006 [12]		X	systematic review
24	Rossignol 2011 [24]	X				Garstang 2006 [12] Wright 2011 [13]		X	systematic review
25	Rossignol 2009 [25]	X				Garstang 2006 [12]		X	systematic review (321 references)
26	Wirojanan 2009 [26]	X						X	RCT **without subgroup analyses**
**MEDLINE**									
1	Williamson 2017 [27]		X			Wright 2011 [13], Cortesi 2012 [14]		X	review
2	´Appendix D, Risk of Bias Ratings Table D-1 Risk of bias assessments´, in: Williamson 2017 [27]							X	review
3	´Appendix D, Excluded Studies´, in: [28]					Wright 2011 [13], Cortesi 2012 [14]		X	review
4	´Appendix B, List of Excluded and							X	systematic review 10)

	Background Studies´, in: Peterson 2024 [29]								
5	´Appendix C Excluded Studies´, in: Williamson 2017 [27]							X	review
6	Scottish guideline 2007 [30]							X	review
7	Scottish guideline 2016 [31]					Cortesi 2012 [14]		X	review
8	Treadwell 2022 [32]							X	review
9	´Applicability Summary Tables´, in: Williamson 2017 [27]							X	review
10	´List of Included Studies´, in: Peterson 2024 [29]							X	systematic review
11	´Appendix K Excluded Studies´, in: National Guideline Alliance (UK) 2017 [33]							X	review 11)
12	´Diskussion´, in: Williamson 2017 [27]							X	review 12)
13	´Results´, in: Williamson 2017 [27]					Cortesi 2012 [14]		X	review
14	Pillay 2021 [34]							X	review
15	Saldanha 2020 [35]							X	review

16	Nowaczyk 2020 [36]							X	review
17	Viswanathan 2020 [37]							X	review
18	Hunter 2024 [38]							X	review
19	Wagner 2019 [39]							X	review
20	Mesh (Medidal Subject Headings)							X	classification only 13)
21	Scottish guideline 2021 [40]							X	review
22	Oral Health 2000 [41]							X	review
23	Parvataneni 2020 [8]							X	systematic review
24	Malow 2021 [42]							X	open label
25	Maras 2018 [17]							X	open label
**PsycINFO** (https://www.apa.org/search?query=melatonin%20autism%20randomized)									
1	Pujol LA 2022 [43]							X	viloxazine for ADHD
2	Webb 2015 [44]							X	review
3	Abrams 2020 [45]							X	review
**Cochrane CENTRAL**									
1	Neurim 2020 [46]								´Withdrawn: **The study stopped** early, before enrolling ist first participant´, ´**Study was not initiated** it was decided to cancel this study.
2	Tordjman 2017 [47]							X	review
3	Tse 2022 [48]							X	**Effects of physical exercise** on sleep, melatonin level, and behavioral functioning in children with autism
4	Jain 2015 [49]							X	In Cochrane CENTRAL protocol only, pilot study of melatonin and **epilepsy** 14)
**5**	**Hayashi 2022 [6]**	**X**					**X**		**RCT, see Tab. 1**

**6**	**Wright 2011 [50]**	**X**					**X**		**RCT, see Tab. 1**
7	Tse 2022 [48]							X	double, see above Cochrane CENTRAL result No. 3
8	Almirall 2012 [51]							X	only design of pilot studies
9	Yuge 2020 [52]							X	only in open label design, RCT was planned, see NCT02757066, 2016)
10	Tse 2018 [53]							X	only study protocol
11	Gringras 2017 [18]							X	without subgroup analysis (ASD, ADHD, Smith-Magenis-Syndrome)
12	Tordjman 2013 [54]							X	only protocol
13	Wirojanan 2009 [26]							X	without subgroup analysis
14	Malow 2012 [22]							X	open label
15	Buratto 2023, see 15)							X	Study of metabolic effects 15)
16	Tse 2022 [4]							X	only study protocol
17	Gringras 2017 [18], Malow 2021 [42]							X	double, see above Cochrane CENTRAL result No. 11; Malow 2021 = open label
**18**	**Garstang 2006 [12]**	**X**			**X**		**X**		**RCT, see Tab. 1**
19	Nir 2019 [55]							X	= Nir 2017 only poster abstract
20	Schroder 2019 [10]							X	without subgroup analysis
21	Maras 2018 [17]							X	open label
**22**	**Wright 2011 [13]**	**X**			**X**		**X**		**RCT, see Tab. 1**
23	Ratliff-Schaub 2005 [56]							X	transdermal secretin
24	Schröder 2022 [5]							X	review
**25**	**Tse 2024 [57]**				**X**		**X**		**RCT, see Tab. 1**
26	Ayuse 2020 [58]							X	effect of ramelteon; study protocol
27	Findling 2017 [59]								abstract

28	Gringras 2017 [60]								abstract
**29**	**Cortesi 2012 [14]**	**X**			**X**		**X**		**RCT, see Tab. 1**
30	Gringras 2017 [61]							X	abstract
31	Nir 2019 [62]							X	abstract
32	Wasdell 2008 [63]							X	without subgroup analysis
33	Dean 2017 [64]							X	N-acetyl cysteine
34	Wink 2018 [65]							X	riluzole
35	Raghavan 2023 [3]							X	Gut and Beta 1,3-1,6 Glucans
36	Sprengers 2019 [66]							X	bumetanide, abstract
37	Aman 2015 [67]							X	risperidone
38	Ghuman 2009							X	methylphenidate
39	Lewis/Papadopoulos 2024 [68]							X	telehealth
40	„early termination“ 16)							X	bumetanide
41	Papadopoulos 2024 [69]							X	sleeping sound/ behavioural sleep intervention program
42	Hannant 2021 [70]							X	Oolong tea (GABA tea)

1) Persico et al: 2025: „Melatonin and oxytocin were not included, because recent systematic reviews have been already published for both these compounds.“[1]. In Part I of this review, the authors look at atypical antipsychotics such as risperidone and aripiprazole, tricyclic antidepressants, SSRIs, especially fluoxetine and sertraline. ‘Mirtazapine helps better with sleep problems.’[71]. Melatonin is not considered in both reviews.
2) 1) The authors cite the reviews by Tordjman et al. 2017 and Rossignol et al. 2011, which are also included in this systematic review. The studies by Gringras et al. 2012 and 2017, Maras et al. 2018, Malow et al. 2020 and Schröder et al. 2019 are mentioned, but not criticised with regard to the lack of subgroup analysis. With regard to pharmacokinetic data, reference is made to the EMA authorisation for slenyto, which is assessed in this systematic review. The study by Wirojanan et al. 2006 is mentioned, but not assessed with regard to the lack of subgroup analyses. The RCT by Wright et al. and the pharmacokinetic study by Goldman et al. 2014 are considered in Schröder et al. 2022 and also in this systematic review.
3) Comparable baseline data as in the review by Schröder et al. 2022 [5], see footnote 2) above for this table.
4) Systematic review in which fewer RCTs were included than in the present publication.

5) Very broad search strategy on the topic ‘Oral melatonin for non-respiratory sleep disturbance in children with neurodisabilities: systematic review and meta-analysis’, which confirmed the published RCTs on the topic of this systematic review. The search strategy is remarkable: ´Databases searched included Applied Social Science Abstracts & Indexes (ASSIA); the Cochrane Central Register of Controlled Trials (CENTRAL); Cochrane Database of Systematic Reviews (CDSR); Conference Proceedings Citation Index; Cumulative Index to Nursing & Allied Health (CINAHL); Database of Abstracts of Reviews of Effects (DARE); Embase; Health Management Information Consortium (HMIC); MEDLINE; MEDLINE In-Process; PsycINFO; Science Citation Index; Social Care Online; and Social Policy & Practice. Searches were undertaken in February and March 2016, and updated in February 2017. The database ClinicalTrials.gov, the World Health Organization’s International Clinical Trials Registry Platform (ICTRP), and the UK Clinical Trials Gateway were also searched for ongoing and completed trials. We reviewed the reference lists of relevant systematic reviews and included studies. Searches were not limited by date, language, or study design.´[11].
6) No evidence for melatonin in ASD in relation to sleep disturbances, not even in the RCT by Mostafavi 2017 cited in this article [72].
7) Systematic review of RCTs on the pharmacological treatment of sleep disorders in childhood and adolescence including reference to 19 RCTs on melatonin, including RCTs on sleep disorders in ASD in childhood and adolescence [16]. The cited review on autism by Lauritsen et al. 2013 does not refer to RCTs on melatonin [73]. In the cited systematic review with derivation of a guideline, in which expert opinions were also included, by Malow et al. 2012: ´A practice pathway for the identification, evaluation, and management of insomnia in children and adolescents with autism spectrum disorders´ [74] the following databases were searched for English-language studies from the years 1995 to 2010: ´OVID, CINAHL, Embase, Database of Abstracts and Review Database of Abstracts of Reviews and Effects, and the Cochrane Database of Systematic reviews databases´[74]. An interesting ‘practice pathway for insomnia in children who have ASD’ was derived from this [74]. However, only studies with more than 10 patients were included, so that 20 studies were considered from 1528 hits - including 3 RCTs by Wright et al. 2011 [13], Garstang et al. 2006 [12], Wirojanan et al. 2009 *without subgroup analysis* [26].
8) Review focussing on complementary medicine treatments for children with ASD [19]. Therein reference to Malow et al. 2012 [22], ´an **open-label** dose-escalation study´[22].
9) Parvataneni et al. 2020 [8].
10) Reference to Niederhofer et al. 2003 [75]: ´Brief Report: Melatonin facilitates sleep in individuals with mental retardation and insomnia.´, RCT, N=20 (IQ<70, 14-18 years, non retarded melatonin gelatine capsules 01.1 or 0.3mg for 7 days, monitored polysomnographically on the last three nights of each treatment wee (days 5-7) and blood samples every 15-60 minutes from 17:00h to 17:00h): ´mentally retarded insomniacs responded to a physiologic melatonin dose (0.3mg) with a statistically significant improvement in sleep quality and without subsequent alterations in the normal circadian pattern of circulating melatonin levels“ [75].
11) p. 88: ´Melatonin. One review included 18 studies addressing melatonin for the treatment of sleep problems in ASD.[147] Thirteen observational studies reported improvements in sleep duration, night awakenings, or sleep onset latency, as did five RCTs. Investigators meta-analyzed RCT data and reported significant improvements on these measures with melatonin versus placebo. Harms associated with melatonin included drowsiness, gastrointestinal symptoms, and worsening behavior. No study reported serious adverse events.“
[147] = Roosignol et al. 2011 [24].
12) https://id.nlm.nih.gov/mesh/D001321.html

13) The resulting publication as an RCT with a significant influence of melatonin on sleep parameters in children with epilepsy has been included in the review of RCTs on melatonin in children and adolescents with non-organic sleep disorders [76].
14) To date only available as a dissertation with English abstract: https://repositorio.unifesp.br/items/8e41030d-8e74-4b0c-9793-59daa685ae15
15) Early Termination BUMAUTEP – EudraCT No: 2016-000106-11, see: https://www.clinicaltrialsregister.eu/ctr-search/trial/2016-000106-11/results

[1] Persico AM, Asta L, Chehbani F, Mirabelli S, Parlatini V, Cortese S, Arango C, Vitiello B: The pediatric psychopharmacology of autism spectrum disorder: A systematic review - Part II: The future. Progress in neuro-psychopharmacology & biological psychiatry 2025;136:111176.

[2] Liang X, Haegele JA, Tse AC, Li M, Zhang H, Zhao S, Li SX: The impact of the physical activity intervention on sleep in children and adolescents with autism spectrum disorder: A systematic review and meta-analysis. Sleep medicine reviews 2024;74:101913.

[3] Raghavan K, Dedeepiya VD, Yamamoto N, Ikewaki N, Sonoda T, Iwasaki M, Kandaswamy RS, Senthilkumar R, Preethy S, Abraham SJK: Benefits of Gut Microbiota Reconstitution by Beta 1,3-1,6 Glucans in Subjects with Autism Spectrum Disorder and Other Neurodegenerative Diseases. Journal of Alzheimer's disease : JAD 2023;94:S241-s252.

[4] Tse ACY, Lee PH, Lau EYY, Cheng JCH, Ho AWY, Lai EWH: Study protocol for a randomized controlled trial comparing the effectiveness of physical exercise and melatonin supplement on treating sleep disturbance in children with autism spectrum disorders. PloS one 2022;17:e0270428.

[5] Schröder CM, Broquère MA, Claustrat B, Delorme R, Franco P, Lecendreux M, Tordjman S: [Therapeutic approaches for sleep and rhythms disorders in children with ASD]. L'Encephale 2022;48:294-303.

[6] Hayashi M, Mishima K, Fukumizu M, Takahashi H, Ishikawa Y, Hamada I, Sugioka H, Yotsuya O, Yamashita Y: Melatonin Treatment and Adequate Sleep Hygiene Interventions in Children with Autism Spectrum Disorder: A Randomized Controlled Trial. Journal of autism and developmental disorders 2022;52:2784-2793.

[7] Zisapel N: Assessing the potential for drug interactions and long term safety of melatonin for the treatment of insomnia in children with autism spectrum disorder. Expert review of clinical pharmacology 2022;15:175-185.

[8] Parvataneni T, Srinivas S, Shah K, Patel RS: Perspective on Melatonin Use for Sleep Problems in Autism and Attention-Deficit Hyperactivity Disorder: A Systematic Review of Randomized Clinical Trials. Cureus 2020;12:e8335.

[9] Cortese S, Wang F, Angriman M, Masi G, Bruni O: Sleep Disorders in Children and Adolescents with Autism Spectrum Disorder: Diagnosis, Epidemiology, and Management. CNS drugs 2020;34:415-423.

[10] Schroder CM, Malow BA, Maras A, Melmed RD, Findling RL, Breddy J, Nir T, Shahmoon S, Zisapel N, Gringras P: Pediatric Prolonged-Release Melatonin for Sleep in Children with Autism Spectrum Disorder: Impact on Child Behavior and Caregiver's Quality of Life. Journal of autism and developmental disorders 2019;49:3218-3230.

[11] Parker A, Beresford B, Dawson V, Elphick H, Fairhurst C, Hewitt C, Scantlebury A, Spiers G, Thomas M, Wright K, McDaid C: Oral melatonin for non-respiratory sleep disturbance in children with

neurodisabilities: systematic review and meta-analyses. Developmental medicine and child neurology 2019;61:880-890.

[12] Garstang J, Wallis M: Randomized controlled trial of melatonin for children with autistic spectrum disorders and sleep problems. Child: care, health and development 2006;32:585-589.

[13] Wright B, Sims D, Smart S, Alwazeer A, Alderson-Day B, Allgar V, Whitton C, Tomlinson H, Bennett S, Jardine J, et al.: Melatonin versus placebo in children with autism spectrum conditions and severe sleep problems not amenable to behaviour management strategies: a randomised controlled crossover trial. Journal of autism and developmental disorders 2011;41:175-184.

[14] Cortesi F, Giannotti F, Sebastiani T, Panunzi S, Valente D: Controlled-release melatonin, singly and combined with cognitive behavioural therapy, for persistent insomnia in children with autism spectrum disorders: a randomized placebo-controlled trial. Journal of sleep research 2012;21:700-709.

[15] Goltz J, Ivanov I, Rice TR: Second generation antipsychotic-induced weight gain in youth with autism spectrum disorders: a brief review of mechanisms, monitoring practices, and indicated treatments. International journal of developmental disabilities 2019;67:159-167.

[16] McDonagh MS, Holmes R, Hsu F: Pharmacologic Treatments for Sleep Disorders in Children: A Systematic Review. Journal of child neurology 2019;34:237-247.

[17] Maras A, Schroder CM, Malow BA, Findling RL, Breddy J, Nir T, Shahmoon S, Zisapel N, Gringras P: Long-Term Efficacy and Safety of Pediatric Prolonged-Release Melatonin for Insomnia in Children with Autism Spectrum Disorder. Journal of child and adolescent psychopharmacology 2018;28:699-710.

[18] Gringras P, Nir T, Breddy J, Frydman-Marom A, Findling RL: Efficacy and Safety of Pediatric Prolonged-Release Melatonin for Insomnia in Children With Autism Spectrum Disorder. Journal of the American Academy of Child and Adolescent Psychiatry 2017;56:948-957.e944.

[19] Klein N, Kemper KJ: Integrative Approaches to Caring for Children with Autism. Current problems in pediatric and adolescent health care 2016;46:195-201.

[20] Rossignol DA, Frye RE: Melatonin in autism spectrum disorders. Current clinical pharmacology 2014;9:326-334.

[21] Tordjman S, Najjar I, Bellissant E, Anderson GM, Barburoth M, Cohen D, Jaafari N, Schischmanoff O, Fagard R, Lagdas E, Kermarrec S, Ribardiere S, Botbol M, Fougerou C, Bronsard G, Vernay-Leconte J: Advances in the research of melatonin in autism spectrum disorders: literature review and new perspectives. International journal of molecular sciences 2013;14:20508-20542.

[22] Malow B, Adkins KW, McGrew SG, Wang L, Goldman SE, Fawkes D, Burnette C: Melatonin for sleep in children with autism: a controlled trial examining dose, tolerability, and outcomes. Journal of autism and developmental disorders 2012;42:1729-1737; author reply 1738.

[23] Anagnostou E, Hansen R: Medical treatment overview: traditional and novel psycho-pharmacological and complementary and alternative medications. Current opinion in pediatrics 2011;23:621-627.

[24] Rossignol DA, Frye RE: Melatonin in autism spectrum disorders: a systematic review and meta-analysis. Developmental medicine and child neurology 2011;53:783-792.

[25] Rossignol DA: Novel and emerging treatments for autism spectrum disorders: a systematic review. Annals of clinical psychiatry : official journal of the American Academy of Clinical Psychiatrists 2009;21:213-236.

[26] Wirojanan J, Jacquemont S, Diaz R, Bacalman S, Anders TF, Hagerman RJ, Goodlin-Jones BL: The efficacy of melatonin for sleep problems in children with autism, fragile X syndrome, or autism and fragile X syndrome. Journal of clinical sleep medicine : JCSM : official publication of the American Academy of Sleep Medicine 2009;5:145-150.

[27] Williamson E, Sathe NA, Andrews JC, Krishnaswami S, McPheeters ML, Fonnesbeck C, Sanders K, Weitlauf A, Warren Z: AHRQ Comparative Effectiveness Reviews. Medical Therapies for Children With Autism Spectrum Disorder—An Update. Rockville (MD): Agency for Healthcare Research and Quality (US); 2017.

[28] Weitlauf AS, Sathe NA, McPheeters ML, Warren Z: AHRQ Comparative Effectiveness Reviews. Interventions Targeting Sensory Challenges in Children With Autism Spectrum Disorder—An Update. Rockville (MD): Agency for Healthcare Research and Quality (US); 2017.

[29] Peterson BS, Trampush J, Maglione M, Bolshakova M, Brown M, Rozelle M, Motala A, Yagyu S, Miles J, Pakdaman S, Gastelum M, Nguyen BT, Tokutomi E, Lee E, Belay JZ, Schaefer C, Coughlin B, Celosse K, Molakalapalli S, Shaw B, Sazmin T, Onyekwuluje AN, Tolentino D, Hempel S: AHRQ Comparative Effectiveness Reviews. ADHD Diagnosis and Treatment in Children and Adolescents. Rockville (MD): Agency for Healthcare Research and Quality (US); 2024.

[30] Scottish Intercollegiate Guidelines N: Assessment, diagnosis and clinical interventions for children and young people with autism spectrum disorders : a national clinical guideline. Edinburgh, Scotland: SIGN; 2007.

[31] Healthcare Improvement Scotland ib, Scottish Intercollegiate Guidelines Network ib: Assessment, diagnosis and interventions for autism spectrum disorders : a national clinical guideline. Edinburgh, Scotland: Scottish Intercollegiate Guidelines Network SIGN; 2016.

[32] Treadwell JR, Wu M, Tsou AY: AHRQ Comparative Effectiveness Reviews. Management of Infantile Epilepsies. Rockville (MD): Agency for Healthcare Research and Quality (US); 2022.

[33] National Guideline A: National Institute for Health and Care Excellence: Guidelines. Cerebral palsy in under 25s: assessment and management. London: National Institute for Health and Care Excellence (NICE)

Copyright National Institute for Health and Care Excellence 2017.; 2017.

[34] Pillay J, Donovan L, Guitard S, Zakher B, Korownyk C, Gates M, Gates A, Vandermeer B, Bougatsos C, Chou R, Hartling L: U.S. Preventive Services Task Force Evidence Syntheses, formerly Systematic Evidence Reviews. Screening for Gestational Diabetes Mellitus: A Systematic Review to Update the 2014 US Preventive Services Task Force Recommendation. Rockville (MD): Agency for Healthcare Research and Quality (US); 2021.

[35] Saldanha IJ, Roth JL, Chen KK, Zullo AR, Adam GP, Konnyu KJ, Cao W, Bhuma MR, Kimmel HJ, Mehta S, Riester MR, Sorial MN, Balk EM: AHRQ Comparative Effectiveness Reviews. Management of Primary Headaches in Pregnancy. Rockville (MD): Agency for Healthcare Research and Quality (US); 2020.

[36] Nowaczyk MJM, Wassif CA: Smith-Lemli-Opitz Syndrome, Last Update January 30, 2020. In: Adam MP, Feldman J, Mirzaa GM, Pagon RA, Wallace SE, Amemiya A, editors. GeneReviews(®). Seattle (WA): University of Washington, Seattle

Copyright © 1993-2025, University of Washington, Seattle. GeneReviews is a registered trademark of the University of Washington, Seattle. All rights reserved.; 2020.

[37] Viswanathan M, Kennedy SM, McKeeman J, Christian R, Coker-Schwimmer M, Cook Middleton J, Bann C, Lux L, Randolph C, Forman-Hoffman V: AHRQ Comparative Effectiveness Reviews. Treatment of Depression in Children and Adolescents: A Systematic Review. Rockville (MD): Agency for Healthcare Research and Quality (US); 2020.

[38] Hunter JE, Berry-Kravis E, Hipp H, Todd PK: FMR1 Disorders. Updated 2024 May 16. In: Adam MP, Feldman J, Mirzaa GM, Pagon RA, Wallace SE, Amemiya A, editors. GeneReviews((R)). Seattle (WA): University of Washington, Seattle

Copyright © 1993-2025, University of Washington, Seattle. GeneReviews is a registered trademark of the University of Washington, Seattle. All rights reserved.; 2024.

[39] Wagner VF, Northrup H: Mucopolysaccharidosis Type III. Updated 2019 Sep 19. In: Adam MP, Feldman J, Mirzaa GM, Pagon RA, Wallace SE, Amemiya A, editors. GeneReviews(®). Seattle (WA): University of Washington, Seattle

Copyright © 1993-2025, University of Washington, Seattle. GeneReviews is a registered trademark of the University of Washington, Seattle. All rights reserved.; 2019.

[40] Healthcare Improvement Scotland ib, National Health Service in Scotland ib, Scottish Intercollegiate Guidelines Network ib: Epilepsies in children and young people : investigative procedures and management : a national clinical guideline. Edinburgh, Scotland: Scottish Intercollegiate Guidelines Network SIGN; 2021.

[41] National Institute of Dental and Craniofacial Research (U.S.) United States. Public Health Service. Office of the Surgeon General. Oral Health in America: A Report of the Surgeon General https://profilesnlmnihgov/spotlight/nn/catalog/nlm:nlmuid-101584932X142-doc 2000.

[42] Malow BA, Findling RL, Schroder CM, Maras A, Breddy J, Nir T, Zisapel N, Gringras P: Sleep, Growth, and Puberty After 2 Years of Prolonged-Release Melatonin in Children With Autism Spectrum

Disorder. Journal of the American Academy of Child and Adolescent Psychiatry 2021;60:252-261.e253.

[43] Pujol LA: Viloxazine Extended-Release (ER) As A Novel Nonstimulant For Treatment Of Attention-Deficit Hyperactivity Disorder. The Tablet Society for Prescribing psychology 2022;Spring 2022:8-11.

[44] Webb N: Pharmacotherapy for pediatric traumatic brain injury. The Tablet. https://www.apadivisions.org/division-55/publications/tablet/2015/12/pediatric-brain-injury The Tablet Society for Prescribing psychology 2015;December.

[45] Abrams Z: Abrams, Z. (2020, July 1). Helping children get a good night's sleep. Monitor on Psychology. https://www.apa.org/monitor/2020/07/ce-corner-sleep Monitor on Psychology 2020;51.

[46] Neurim Pharmaceuticals Ltd. A Randomized, Placebo Controlled Study to Investigate the Efficacy and Safety of Slenyto® to Alleviate Sleep Disturbances in Children With Autism Spectrum Disorder. Withdrawn 2021/03/12/ . No Results Submitted. NCT04233502. https://clinicaltrials.gov/study/NCT04233502. 2020.

[47] Tordjman S, Chokron S, Delorme R, Charrier A, Bellissant E, Jaafari N, Fougerou C: Melatonin: Pharmacology, Functions and Therapeutic Benefits. Current neuropharmacology 2017;15:434-443.

[48] Tse ACY, Lee PH, Zhang J, Chan RCY, Ho AWY, Lai EWH: Effects of exercise on sleep, melatonin level, and behavioral functioning in children with autism. Autism: the international journal of research & practice 2022;26:1712-1722.

[49] Jain SV, Horn PS, Simakajornboon N, Beebe DW, Holland K, Byars AW, Glauser TA: Melatonin improves sleep in children with epilepsy: a randomized, double-blind, crossover study. Sleep medicine 2015;16:637-644.

[50] Wright B, Sims D, Smart S, Alwazeer A, Alderson-Day B, Allgar V, Whitton C, Tomlinson H, Bennett S, Jardine J, McCaffrey N, Leyland C, Jakeman C, Miles J: Melatonin versus placebo in children with autism spectrum conditions and severe sleep problems not amenable to behaviour management strategies: a randomised controlled crossover trial. Journal of autism and developmental disorders 2011;41:175-184.

[51] Almirall D, Compton SN, Gunlicks-Stoessel M, Duan N, Murphy SA: Designing a pilot sequential multiple assignment randomized trial for developing an adaptive treatment strategy. Statistics in medicine 2012;31:1887-1902.

[52] Yuge K, Nagamitsu S, Ishikawa Y, Hamada I, Takahashi H, Sugioka H, Yotsuya O, Mishima K, Hayashi M, Yamashita Y: Long-term melatonin treatment for the sleep problems and aberrant behaviors of children with neurodevelopmental disorders. BMC psychiatry 2020;20:445.

[53] Tse ACY, Lee PH, Zhang J, Lai EWH: Study protocol for a randomised controlled trial examining the association between physical activity and sleep quality in children with autism spectrum disorder based on the melatonin-mediated mechanism model. BMJ open 2018;8:e020944.

[54] Tordjman S, Kermarrec S, Cohen D, Xavier J, Rolland AC, Bouvet M, Gicquel L, Martin V, Chevreuil C, Seveno T, Botbol M, Touitou Y, Ribardière S, Bellissant E: Meladose : étude de la relation dose–effet de la mélatonine dans l'autisme infantile. Neuropsychiatrie de l'Enfance et de l'Adolescence 2013;61:415-416.

[55] Nir T, Maras A, Malow B, Schroder C, Gringras P, Zisapel N: Pediatric prolonged-release melatonin for sleep in children with autism spectrum disorder: implications for child behavior and caregiver's quality of life. European psychiatry 2019;56:S572.

[56] Ratliff-Schaub K, Carey T, Reeves GD, Rogers MA: Randomized controlled trial of transdermal secretin on behavior of children with autism. Autism : the international journal of research and practice 2005;9:256-265.

[57] Tse ACY, Lee PH, Sit CHP, Poon ET, Sun F, Pang CL, Cheng JCH: Comparing the Effectiveness of Physical Exercise Intervention and Melatonin Supplement in Improving Sleep Quality in Children with ASD. Journal of autism and developmental disorders 2024;54:4456-4464.

[58] Ayuse T, Ozaki-Honda Y, Kurata S, Mishima G, Kiriishi K, Magata N, Kawasaki H, Yamaguchi-Komeyama K, Tanoue N, Ayuse T: Study on the preventive effect of ramelteon on the onset of sleep disorder after general anesthesia in patients with autism spectrum disorder: A study protocol. Medicine 2020;99:e22826.

[59] Findling RL, Gringras P, Nir T, Zisapel N: Conference proceeding. Short- and long-term prolonged release melatonin treatment for sleep disorders in children with autism spectrum disorders - Results of a phase III randomized clinical trial. Journal of the American Academy of Child and Adolescent Psychiatry 2017;56:S167.

[60] Gringras P, Findling RL, Nir T, Zisapel N: Abstract. Conference Proceedings. Short and long term prolonged release melatonin treatment for sleep disorders in children with autism spectrum disorders: results of a phase III randomized clinical trial. Sleep Medicine, Suppl 1 2017;40:e119.

[61] Gringras P, Findling R, Nir T, Zisapel N: Oral presentations (Abstract). Short and Long Term Prolonged Release Melatonin Treatment For Sleep Disorders In Children With Autism Spectrum Disorders. Results Of A Phase III Randomized Clinical Trial. Developmental Medicine & Child Neurology 2017;59:5-38.

[62] Nir T, Maras A, Malow B, Schroder C, Gringras P, Zisapel N: Oral presentation. Abstract. Pediatric prolonged-release melatonin for sleep in children with autism spectrum disorder: implications for child behavior and caregiver's quality of life. European psychiatry 2019;56:S572.

[63] Wasdell MB, Jan JE, Bomben MM, Freeman RD, Rietveld WJ, Tai J, Hamilton D, Weiss MD: A randomized, placebo-controlled trial of controlled release melatonin treatment of delayed sleep phase syndrome and impaired sleep maintenance in children with neurodevelopmental disabilities. Journal of pineal research 2008;44:57-64.

[64] Dean OM, Gray KM, Villagonzalo KA, Dodd S, Mohebbi M, Vick T, Tonge BJ, Berk M: A randomised, double blind, placebo-controlled trial of a fixed dose of N-acetyl cysteine in children with autistic disorder. Australian and New Zealand journal of psychiatry 2017;51:241-249.

[65] Wink LK, Adams R, Horn PS, Tessier CR, Bantel AP, Hong M, Shaffer RC, Pedapati EV, Erickson CA: A Randomized Placebo-Controlled Cross-Over Pilot Study of Riluzole for Drug-Refractory Irritability in Autism Spectrum Disorder. Journal of autism and developmental disorders 2018;48:3051-3060.

[66] Sprengers J, Andel van D, Oranje B, Linkenkaer-Hansen KK, Hilgo B: Abstract. P.4.11. Bumetanide in Autism Medication and Biomarker (BAMBI) study: Medication response profiles. European Neuropsychopharmacology, Suppl 2 2019;29:S708-S709.

[67] Aman M, Rettiganti M, Nagaraja HN, Hollway JA, McCracken J, McDougle CJ, Tierney E, Scahill L, Arnold LE, Hellings J, et al.: Tolerability, Safety, and Benefits of Risperidone in Children and Adolescents with Autism: 21-Month Follow-up After 8-Week Placebo-Controlled Trial. Journal of child and adolescent psychopharmacology 2015;25:482-493.

[68] Lewis S, Rinehart N, Mantilla A, Alvares G, Hiscock H, Marks D, Papadopoulos N: A pilot randomised controlled trial of a telehealth-delivered brief 'Sleeping Sound Autism' intervention for autistic children. Sleep medicine 2024;124:162-173.

[69] Papadopoulos N, Sciberras E, Hiscock H, Williams K, McGillivray J, Mihalopoulos C, Engel L, Fuller-Tyszkiewicz M, Bellows ST, Marks D, Howlin P, Rinehart N: Sleeping Sound Autism Spectrum Disorder (ASD): a randomised controlled trial of a brief behavioural sleep intervention in primary school-aged autistic children. Journal of child psychology and psychiatry, and allied disciplines 2022;63:1423-1433.

[70] Hannant P, Cassidy S, Renshaw D, Joyce A: A double-blind, placebo-controlled, randomised-designed GABA tea study in children diagnosed with autism spectrum conditions: a feasibility study clinical trial registration: ISRCTN 72571312. Nutritional neuroscience 2021;24:45-61.

[71] Persico AM, Ricciardello A, Lamberti M, Turriziani L, Cucinotta F, Brogna C, Vitiello B, Arango C: The pediatric psychopharmacology of autism spectrum disorder: A systematic review - Part I: The past and the present. Progress in neuro-psychopharmacology & biological psychiatry 2021;110:110326.

[72] Mostafavi SA, Solhi M, Mohammadi MR, Akhondzadeh S: Melatonin for Reducing Weight Gain Following Administration of Atypical Antipsychotic Olanzapine for Adolescents with Bipolar Disorder: A Randomized, Double-Blind, Placebo-Controlled Trial. Journal of child and adolescent psychopharmacology 2017;27:440-444.

[73] Lauritsen MB: Autism spectrum disorders. European child & adolescent psychiatry 2013;22:37-42.

[74] Malow BA, Byars K, Johnson K, Weiss S, Bernal P, Goldman SE, Panzer R, Coury DL, Glaze DG: A practice pathway for the identification, evaluation, and management of insomnia in children and adolescents with autism spectrum disorders. Pediatrics 2012;130 Suppl 2:S106-124.

[75] Niederhofer H, Staffen W, Mair A, Pittschieler K: Brief report: melatonin facilitates sleep in individuals with mental retardation and insomnia. Journal of autism and developmental disorders 2003;33:469-472.

[76] Paditz E: Melatonin bei Schlafstörungen im Kindes- und Jugendalter. Monatsschrift Kinderheilkunde 2024;172:44-51.
