# Supplementary material for: The Pharmacokinetics, Dosage, Preparation Forms, and Efficacy of Orally Administered Melatonin for Non-Organic Sleep Disorders in Autism Spectrum Disorder During Childhood and Adolescence: A Systematic Review
Source: Children (Basel). 2025 May 16;12(5):648. doi: 10.3390/children12050648 (PMC12110111; doi:10.3390/children12050648)
Supplement: Supplementary file 1 [file children-12-00648-s001.zip › Supplement 2_Paditz E et al._children 2025_Melatonin Autism Systematic Review_CASP-checklist-randomised-controlled-trials-RCT-2024.pdf]

CASP

Critical Appraisal
Skills Programme

CASP Checklist:
For Randomised Controlled Trials (RCTs)

https://casp-uk.net/casp-tools-checklists/randomised-controlled-trial-rct-checklist/

Supplement 2 to Paditz E et al., children 2025: Melatonin/Autism/systematic review

Reviewer Name:	Ekkehart Paditz
Paper Title:<br>Author:<br>Web Link:<br>Appraisal Date:	Garstang 2006 [1], Wright 2011 [2], Cortesi 2012 [3], Hayashi 2022 [4], Tse 2024 [5]:<br>**[1] Garstang** J, Wallis M: Randomized controlled trial of melatonin for children with autistic spectrum disorders and sleep problems. Child: care, health and development 2006;32:585-589.<br>**[2] Wright** B, et al.: Melatonin versus placebo in children with autism spectrum conditions and severe sleep problems not amenable to behaviour management strategies: a randomised controlled crossover trial. Journal of autism and developmental disorders 2011;41:175-184.<br>**[3] Cortesi** F, et al.: Controlled-release melatonin, singly and combined with cognitive behavioural therapy, for persistent insomnia in children with autism spectrum disorders: a randomized placebo-controlled trial. Journal of sleep research 2012;21:700-709.<br>**[4] Hayashi** M, et al.: Melatonin Treatment and Adequate Sleep Hygiene Interventions in Children with Autism Spectrum Disorder: A Randomized Controlled Trial. Journal of autism and developmental disorders 2022;52:2784-2793.<br>**[5] Tse** ACY, et al.: Comparing the Effectiveness of Physical Exercise Intervention and Melatonin Supplement in Improving Sleep Quality in Children with ASD. Journal of autism and developmental disorders 2024;54:4456-4464.

During critical appraisal, never make assumptions about what the researchers have done. If it is not possible to tell, use the "Can't tell" response box. If you can't tell, at best it means the researchers have not been explicit or transparent, but at worst it could mean the researchers have not undertaken a particular task or process. Once you've finished the critical appraisal, if there are a large number of "Can't tell" responses, consider whether the findings of the study are trustworthy and interpret the results with caution.

Section A Is the basic study design valid for a randomised controlled trial?	
1. Did the study address a clearly formulated research question?<br>2.	☐ Yes ☐ No ☐ Can't Tell<br><br>Yes, for all 5 trials.
CONSIDER:<br>*Was the study designed to assess the outcomes of an intervention?*<br>*Is the research question 'formulated' in terms of:*<br>• *Population studied*<br>• *Intervention given*<br>• *Comparator chosen*<br>• *Outcomes measured?*	
3. Was the assignment of participants to interventions randomised?	☐ Yes ☐ No ☐ Can't Tell<br><br>Yes, for all 5 trials.
CONSIDER:<br>• *How was randomisation carried out? Was the method appropriate?*<br>• *Was randomisation sufficient to eliminate systematic bias?*<br>• *Was the allocation sequence concealed from investigators and participants?*	
4. Were all participants who entered the study accounted for at its conclusion?	☐ Yes ☐ No ☐ Can't Tell<br><br>No, the drop-out rates are given in Table 1b for each study.
CONSIDER:<br>• *Were losses to follow-up and exclusions after randomisation accounted for?*<br>• *Were participants analysed in the study groups to which they were randomised (intention-to-treat analysis)?*<br>• *Was the study stopped early? If so, what was the reason?*	
**Section B Was the study methodologically sound?**	
5. (a) Were the participants 'blind' to intervention they were given?	☐ Yes ☐ No ☐ Can't Tell<br><br>Yes, for all 5 trials.
(b) Were the investigators 'blind' to the intervention they were giving to participants?	☐ Yes ☐ No ☐ Can't Tell<br><br>Yes, for all 5 trials.
(c) Were the people assessing/analysing outcome/s 'blinded'?	☐ Yes ☐ No ☐ Can't Tell

	Yes, for all 5 trials.
6. Were the study groups similar at the start of the randomised controlled trial?	☐Yes ☐ No ☐ Can't Tell<br><br>Yes, for all 5 trials.<br>The primary group sizes changed due to the drop outs declared in each study. In the RCTs with cross-over design, the groups were not changed.
CONSIDER:<br>• *Were the baseline characteristics of each study group (e.g. age, sex, socio-economic group) clearly set out?*<br>• *Were there any differences between the study groups that could affect the outcome/s?*	
7. Apart from the experimental intervention, did each study group receive the same level of care (that is, were they treated equally)?	☐Yes ☐ No ☐ Can't Tell<br><br>Yes, for all 5 trials.
CONSIDER:<br>• *Was there a clearly defined study protocol?*<br>• *If any additional interventions were given (e.g. tests or treatments), were they similar between the study groups?*<br>• *Were the follow-up intervals the same for each study group?*	
Section C: What are the results?	
8. Were the effects of intervention reported comprehensively?	☐Yes ☐ No ☐ Can't Tell<br><br>If power calculations were performed, it has been indicated in Tab. 1b.<br><br>In all 5 studies, clear outcomes were defined in advance and suitable parameters were declared, see Tab. 1b including the detailed explanations in the footnotes of this table.<br><br>Missings were declared. In our view, the drop-out rates were acceptable and low, so that we do not believe that any serious bias in the results can be assumed.<br><br>The statistical methods used were appropriate; p-values were reported in all studies.
CONSIDER:<br>• *Was a power calculation undertaken?*<br>• *What outcomes were measured, and were they clearly specified?*<br>• *How were the results expressed? For binary outcomes, were relative and absolute effects reported?*<br>• *Were the results reported for each outcome in each study group at each follow-up interval?*<br>• *Was there any missing or incomplete data?*<br>• *Was there differential drop-out between the study groups that could affect the results?*<br>• *Were potential sources of bias identified?*<br>• *Which statistical tests were used?*<br>• *Were p values reported?*	
9. Was the precision of the estimate of the intervention or treatment effect reported?	☐Yes ☐ No ☐ Can't Tell

	Yes, in all 5 trials, as the results were given in absolute and relative frequencies with the corresponding p-values.<br>In view of the limited group sizes, it is understandable from our point of view that only p-values, but no confidence intervals, were determined. Mean values or median values and ranges were specified.
CONSIDER:<br>• *Were confidence intervals (CIs) reported?*	
10. Do the benefits of the experimental intervention outweigh the harms and costs?	☐ Yes ☐ No ☐ Can't Tell<br><br>Yes, for all 5 trials.
CONSIDER:<br>• *What was the size of the intervention or treatment effect?*<br>• *Were harms or unintended effects reported for each study group?*<br>• *Was a cost-effectiveness analysis undertaken? (Cost-effectiveness analysis allows a comparison to be made between different interventions used in the care of the same condition or problem.)*	
Section D: Will the results help locally?	
11. Can the results be applied to your local population/in your context?	☐ Yes ☐ No ☐ Can't Tell<br><br>Yes, for all 5 trials.
CONSIDER:<br>• *Are the study participants similar to the people in your care?*<br>• *Would any differences between your population and the study participants alter the outcomes reported in the study?*<br>• *Are the outcomes important to your population?*<br>• *Are there any outcomes you would have wanted information on that have not been studied or reported?*<br>• *Are there any limitations of the study that would affect your decision?*	
12. Would the experimental intervention provide greater value to the people in your care than any of the existing interventions?	☐ Yes ☐ No ☐ Can't Tell<br><br>Yes, in relation to all 5 RCTs, because they focus on ASD without comorbidities - OR because subgroup analyses were performed if comorbidities were present.
CONSIDER:<br>• *What resources are needed to introduce this intervention taking into account time, finances, and skills development or training needs?*<br>• *Are you able to disinvest resources in one or more existing interventions in order to be able to re-invest in the new intervention?*	

**APPRAISAL SUMMARY**: *List key points from your critical appraisal that need to be considered when assessing the validity of the results and their usefulness in decision-making.*		
**Positive/Methodologically sound**	**Negative/Relatively poor methodology**	**Unknowns**
**These 5 RCTs indicate that oral administration of melatonin in rapid-release non-retarded preparations is effective in children and adolescents with ASD and non-organic sleep disorders and has not shown significant side effects. The limited pharmacokinetic data available to date in children and adolescents with ASD and pathophysiological data support this conclusion. Overall, this provides a well-documented database which can be used as a basis for advising low doses of melatonin with rapid-release preparations in these individuals.**<br>**It is particularly interesting that two RCTs demonstrated combined effects of melatonin in conjunction with cognitive behavioural therapy and in conjunction with physical activation (Cortesi et al. and Tse et al.).**	**To date, there are no RCTs in which slow-release and non-released melatonin preparations have been compared in terms of their efficacy in insomnia. The rapidly increasing melatonin concentrations in the cerebrospinal fluid of the ventricular system during darkness are of major importance for the efficacy of melatonin in the brain. Several animal studies and some data from children and adults are available. This pool of data should be given greater consideration and expanded. The relationships between temperature regulation, sleep, movement and light should be given greater consideration and investigated in future.**<br><br>**In the interesting study by Tse et al. it was unfortunately not recorded a) at what time of day and in what lighting conditions the physical training took place and b) whether the physical training was associated with changes in body weight or metabolic parameters.**	**The metabolic degradation pathways of melatonin in all age groups should be investigated and considered more intensively in the future.**

Referencing recommendation:
CASP recommends using the Harvard style referencing, which is an author/date method. Sources are cited within the body of your assignment by giving the name of the author(s) followed by the date of publication. All other details about the publication are given in the list of references or bibliography at the end.
Example: *Critical Appraisal Skills Programme (2024). CASP (insert name of checklist i.e. randomised controlled trials (RCTs) Checklist.) [online] Available at: insert URL. Accessed: insert date accessed.*
Creative Commons ©CASP this work is licensed under the Creative Commons Attribution – Non-Commercial- Share A like. To view a copy of this licence, visit https://creativecommons.org/licenses/by-nc-sa/4.0/

[1] Garstang J, Wallis M: Randomized controlled trial of melatonin for children with autistic spectrum disorders and sleep problems. Child: care, health and development 2006;32:585-589.

[2] Wright B, Sims D, Smart S, Alwazeer A, Alderson-Day B, Allgar V, Whitton C, Tomlinson H, Bennett S, Jardine J, et al.: Melatonin versus placebo in children with autism spectrum conditions and severe sleep problems not amenable to behaviour management strategies: a randomised controlled crossover trial. Journal of autism and developmental disorders 2011;41:175-184.

[3] Cortesi F, Giannotti F, Sebastiani T, Panunzi S, Valente D: Controlled-release melatonin, singly and combined with cognitive behavioural therapy, for persistent insomnia in children with autism spectrum disorders: a randomized placebo-controlled trial. Journal of sleep research 2012;21:700-709.

[4] Hayashi M, Mishima K, Fukumizu M, Takahashi H, Ishikawa Y, Hamada I, Sugioka H, Yotsuya O, Yamashita Y: Melatonin Treatment and Adequate Sleep Hygiene Interventions in Children with Autism Spectrum Disorder: A Randomized Controlled Trial. Journal of autism and developmental disorders 2022;52:2784-2793.

[5] Tse ACY, Lee PH, Sit CHP, Poon ET, Sun F, Pang CL, Cheng JCH: Comparing the Effectiveness of Physical Exercise Intervention and Melatonin Supplement in Improving Sleep Quality in Children with ASD. Journal of autism and developmental disorders 2024;54:4456-4464.
